# Elesclomol-induced increase of mitochondrial reactive oxygen species impairs glioblastoma stem-like cell survival and tumor growth

**DOI:** 10.1186/s13046-021-02031-4

**Published:** 2021-07-12

**Authors:** Mariachiara Buccarelli, Quintino Giorgio D’Alessandris, Paola Matarrese, Cristiana Mollinari, Michele Signore, Andrea Cappannini, Maurizio Martini, Pierluigi D’Aliberti, Gabriele De Luca, Francesca Pedini, Alessandra Boe, Mauro Biffoni, Roberto Pallini, Lucia Ricci-Vitiani

**Affiliations:** 1 Department of Oncology and Molecular Medicine, Istituto Superiore di Sanità, Viale Regina Elena 299, 00161 Rome, Italy; 2 Fondazione Policlinico Universitario A. Gemelli IRCCS, Roma, Italy; 3 Institute of Neurosurgery, Catholic University School of Medicine, Rome, Italy; 4 Center for Gender Specific Medicine, Istituto Superiore di Sanità, Rome, Italy; 5 Institute of Translational Pharmacology, National Research Council, Rome, Italy; 6 Department of Neuroscience, Istituto Superiore di Sanità, Rome, Italy; 7 Core Facilities, Istituto Superiore di Sanità, Rome, Italy; 8 Sapienza, University of Rome, Rome, Italy; 9 Institute of Pathology, Catholic University School of Medicine, Rome, Italy

**Keywords:** Glioblastoma, Cancer stem cells, Elesclomol, Oxidative stress

## Abstract

**Background:**

Glioblastoma (GBM) is the most common and aggressive primary malignant brain tumor in adults, characterized by a poor prognosis mainly due to recurrence and therapeutic resistance. It has been widely demonstrated that glioblastoma stem-like cells (GSCs), a subpopulation of tumor cells endowed with stem-like properties is responsible for tumor maintenance and progression. Moreover, it has been demonstrated that GSCs contribute to GBM-associated neovascularization processes, through different mechanisms including the transdifferentiation into GSC-derived endothelial cells (GdECs).

**Methods:**

In order to identify druggable cancer-related pathways in GBM, we assessed the effect of a selection of 349 compounds on both GSCs and GdECs and we selected elesclomol (STA-4783) as the most effective agent in inducing cell death on both GSC and GdEC lines tested.

**Results:**

Elesclomol has been already described to be a potent oxidative stress inducer. In depth investigation of the molecular mechanisms underlying GSC and GdEC response to elesclomol, confirmed that this compound induces a strong increase in mitochondrial reactive oxygen species (ROS) in both GSCs and GdECs ultimately leading to a non-apoptotic copper-dependent cell death. Moreover, combined in vitro treatment with elesclomol and the alkylating agent temozolomide (TMZ) enhanced the cytotoxicity compared to TMZ alone. Finally, we used our experimental model of mouse brain xenografts to test the combination of elesclomol and TMZ and confirmed their efficacy in vivo.

**Conclusions:**

Our results support further evaluation of therapeutics targeting oxidative stress such as elesclomol with the aim of satisfying the high unmet medical need in the management of GBM.

**Supplementary Information:**

The online version contains supplementary material available at 10.1186/s13046-021-02031-4.

## Background

Glioblastoma (GBM) is one of the most frequent and lethal type of brain cancer in adults with a median survival of only 14.6 months and less than 5% of patients surviving 5 years after diagnosis [1]. GBM carries a subpopulation of tumor initiating stem-like cells (glioblastoma stem-like cells, GSCs) that has the ability to sustain malignant properties, including initiation, growth, therapy resistance, recurrence and, consequently, should represent a primary therapeutic target [2]. Angiogenesis also plays a significant role in GBM pathobiology mainly because the tumor poses a considerable metabolic demand for nutrients and oxygen delivery to sustain its high rate of cell proliferation and metabolism. GBMs are characterized by increased microvascular proliferation, which is essential for tumor growth and invasion, that is mainly mediated by vascular endothelial growth factor A (VEGF-A) signaling [3, 4]. VEGF is highly expressed in both endothelial and tumor cells and it is transcriptionally activated by hypoxia-inducible factor-1 (HIF-1) [5]. VEGF is also produced by GSCs and transported in extracellular vesicles in the perivascular niche, where it contributes to promote GSC survival and resistance to therapy [6–8]. As VEGF expression increases concomitantly with glioma grade, higher VEGF levels are associated with poor outcome [9, 10] and are found increased in recurrent compared with primary tumor [11]. Anti-angiogenic therapy for GBM has been developed prominently in the form of bevacizumab, a recombinant human anti-VEGF-A monoclonal antibody that acts primarily by neutralizing VEGF to block its binding to VEGF receptors on endothelial cells. Bevacizumab became the first anti-angiogenic treatment to be approved for use in cancer [12]. Clinical trials suggesting a substantial efficacy of bevacizumab in recurrent GBM were reported as early as 2009 [13]. In GBM, bevacizumab has been shown to induce dramatic reductions in tumor contrast enhancement and to improve the time to progression when administered either alone or in combination with chemotherapy [14]. However placebo-controlled trials, while confirming the increase of progression-free survival (PFS), failed to demonstrate an effect of bevacizumab on overall survival (OS) [15–18]. Moreover, while bevacizumab initially results in a biological response, treatment does not prevent aggressive local and diffuse spread [19] and may trigger a phenotypic change in GBM, which acquires a gliomatosis-like growth pattern, the so-called infiltrative shift [20].

We recently investigated the infiltrative growth pattern induced by bevacizumab treatment using both a human specimen and rat models [21]. In the human specimen, a substantial fraction of infiltrating tumor cells is located along perivascular spaces in close relationship with endothelial cells. Infiltrating tumor cells showed tropism for vascular structures and propensity to form tubules and niches with endothelial cells. Molecularly, bevacizumab triggered an epithelial to mesenchymal transition with over-expression of the receptor Plexin Domain Containing 1 (PLXDC1) [21].

Furthermore, we demonstrated that GSCs are able to transdifferentiate into functional endothelial-like cells [22]. Selective targeting of the GSC-derived endothelial cells (GdECs) in xenografts resulted in tumor reduction and degeneration, indicating their functional relevance [22]. The ability of GSCs to directly contribute to the tumor vasculature by endothelial transdifferentiation, represents a new mechanism of angiogenesis. GdECs have the mutations of the parental tumor and, in addition are resistant to traditional antiangiogenic therapies. In a 3D computational model, anti-mitotic and anti-angiogenic targeting result in slow down of tumor growth but increased invasiveness [23], as expected. However, anti-GSC therapies, which promote differentiation or disturb the stem cell niche, reduce tumor invasiveness but are ultimately limited in reducing tumor size because the transdifferentiated cells maintain the GSC population [23]. Then, regimens targeting both GSCs and transdifferentiated GSCs potentially lead to tumor eradication.

With the aim of identifying compounds able to counteract the survival pathways of both GSCs and GdECs, here we have screened an anti-cancer drug library and evaluated the effect on cellular signaling pathways by a (phospho-)proteomic analysis (Reverse-Phase Protein Arrays, RPPA).

## Methods

### Cell cultures

GSCs were isolated from surgical samples of adult patients who underwent craniotomy at the Institute of Neurosurgery, Catholic University of Rome, upon approval by the local ethical committee. Informed consent was obtained from the patients before surgery. After mechanical dissociation, single cell suspension was cultured in a serum-free medium supplemented with epidermal growth factor (EGF) and basic fibroblast growth factor (b-FGF), as previously described [24, 25]. GSC lines were validated by Short Tandem Repeat (STR) DNA fingerprinting. Nine highly polymorphic STR loci plus amelogenin (Cell ID™ System, Promega Inc., Madison, WI, USA) were used [26]. All GSC profiles were challenged against public databases to confirm authenticity [26].

The in vivo tumorigenic potential of GSCs was assayed by intracranial cell injection into immunocompromised mice, resulting in tumors with the same antigen expression and histological tissue organization as the human parent tumor [25, 27]. Human microvascular endothelial cells (HMVECs) were purchased from Lonza and cultured in endothelial basal medium (EBM-2, Lonza Walkersville Inc., Walkerswille, MD, USA) supplemented with EGM™-2 MV SingleQuots™ Kit (Lonza Walkersville Inc.).

The U87MG human GBM cell line was purchased from the American Type Culture Collection (Manassas, VA).

### Transdifferentiation of GSCs

GSCs were cultured in medium supplemented with EGM™-2MV SingleQuots™ Kit (Lonza Walkersville Inc.) and 12 μg/ml Bovine Brain Extract (BBE, Lonza Walkersville Inc.) on Matrigel® (Corning, New York, NY, USA) coated tissue culture surface under hypoxic condition (1% O_2_) for two or 3 weeks. Under these conditions, GSCs grow as continuous net-like structures.

Glial differentiation was obtained by culturing GSCs in stem cell medium supplemented with 10% FBS on Matrigel® (Corning) coated tissue culture surface for 3 weeks.

### Immunostaining

For the expression of the endothelial markers, cells were incubated for 90 min at 4 °C with the antibodies, then washed with PBS and analyzed by the flow cytometer FACSCanto (Becton Dickinson). The antibodies used were as follows: phycoerythrin (PE)-conjugated mouse anti-human CD31 antibody (1:20, BD Biosciences, Milan, Italy); PE-conjugated mouse anti-human CD34 antibody (1:20, clone BIRMA-K3, DakoCytomation, Denmark); PE-conjugated mouse anti-human CD133/1 antibody (1:20, clone AC133, Miltenyi Biotec Inc., Bergisch Gladbach, Germany); PE-conjugated mouse anti-human Tie2 antibody (1:25, R&D Systems, Minneapolis, MN, USA); PE-conjugated mouse anti-human VEGFR2 (KDR) antibody (1:25, R&D Systems) or PE-conjugated mouse IgG_1_ isotype control antibody (Miltenyi Biotec Inc.). Data were analyzed with FACS Diva software (Becton Dickinson).

### Subcutaneous implantation of GdECs and immunohistochemical analysis of tumor

Experiments involving animals were approved according to the Italian law (D. Lgs. 26/2014). After 2 weeks in endothelial culture conditions, GdECs were incubated for 90 min at 4 °C with PE-conjugated mouse anti-human CD34 antibody (1:20, clone BIRMA-K3, DakoCytomation) or PE-conjugated mouse IgG_1_ isotype control antibody (Miltenyi Biotec Inc.). Two subpopulations with different CD34 expression levels were isolated by using FACSAria cell sorter (BD Biosciences). Athymic mice (4–6 weeks old; Charles River, Milan, Italy) were implanted subcutaneously with 2 × 10^5^ CD34^high^ or CD34^−/low^ GdECs. Briefly, 4-μm sections were obtained from formalin-fixed, paraffin-embedded (FFPE) blocks and after antigen retrieval, were deparaffinized, rehydrated and incubated for 1 h at room temperature with a prediluted mouse anti-human CD34 antibody (1:20, clone BIRMA-K3, DakoCytomation) or a prediluted rabbit anti-GFAP monoclonal antibody (Clone EP672Y; Ventana Inc. Tucson, AZ, USA).

### Drug cytotoxicity experiments and in vitro cell treatments

For cytotoxicity experiments, GSCs were mechanically dissociated and plated in a 96-well plate, in triplicate, at a density of 2 × 10^4^ cells/ml. GdECs, glial differentiated cells, U87MG and HMVECs were plated at a density of 1 × 10^4^ cells/ml. Temozolomide (TMZ) was purchased from Sigma (Sigma-Aldrich, St. Louis, MO, USA) and used at the concentration of 450 μM, elesclomol and the anti-cancer compound library were purchased from SelleckChem (Selleck chemicals, Houston, TX, USA). A list of compounds used for the library screening is available at Supplementary Table 1. Compounds were dissolved in DMSO and added 24 h after cell plating at indicated concentrations. After treatment, ATP levels were measured using the CellTiter-Glo™ (Promega Inc.) according to the manufacturer’s instructions. The mean of the raw luminescence values (L_D_) from triplicate wells treated with vehicle alone (DMSO 0.2%, mL_C_), was used as reference to calculate percent viability from wells treated with drugs (V_D_), using the following formula: V_D_ = (L_D_/mL_C_)*100, as previously described [28]. To evaluate synergy between elesclomol and TMZ we used the Bliss additivism model [29] that predicts the combined response *C* for two single compounds with effects *A* and *B* as follows: C = A + B – AxB, where each effect is expressed as percent residual viability.

### RPPA

Protein extracts for RPPA analysis were prepared as previously described [30]. Briefly, 6, 16, 24 and 36 h after treatment, cells were collected, washed twice in PBS and lysed in a home-made buffer containing T-PER reagent (Thermo Fisher Scientific, Waltham, MA, USA), 300 mM NaCl, protease and phosphatase inhibitors cocktails (Merck-Millipore, Burlington, MA, USA). Total protein concentration was measured using the Bradford reagent method (Bio-Rad Laboratories, Hercules, CA, USA). RPPA-ready protein extracts were prepared by diluting lysates in extraction buffer containing 47.5% T-PER, 50% 2X Sodium dodecyl sulfate (SDS) (Thermo Fisher Scientific) and 2.5% Tris (2-carboxyethyl) phosphine hydrochloride (TCEP) (Thermo Fisher Scientific) to a final concentration of 1 mg/mL in a volume of 40 mL. A further denaturation step of 5 min boiling was performed prior to freezing at − 80 °C. RPPA analysis was performed on a per service basis by the MD Anderson Cancer Center RPPA Core Facility, following their standard operating procedures [https://www.mdanderson.org/research/research-resources/core-facilities/functional-proteomics-rppa-core/education-and-references.html]. The list of antibodies utilized for RPPA analysis is available in Supplementary Table 2.

### Cell death evaluation

GSCs were mechanically dissociated and plated at 10 × 10^4^ cells/well in 6-well plates. Chemical treatments were performed following the same protocol of cytotoxicity experiments. The following chemicals were used: 10 μM z-VAD-FMK (Enzo Life Sciences, Rome, Italy), 2 μm ferrostatin-1 (Sigma-Aldrich), 20 μm necrostatin-1 (Enzo Life Sciences), 10 μm CoQ, 5 and 10 mM NAC (Sigma-Aldrich), 10 mM 3-MA (Sigma-Aldrich), 10 μm TTM (Sigma-Aldrich).

### Fluorimetric and flow cytometry evaluations

#### Mitochondrial membrane potential

The mitochondrial membrane potential of controls and treated cells were studied by using Tetramethylrhodamine ester 1 μM (TMRM; Molecular Probes, Eugene, OR, USA). 5–5′,6–6′-tetrachloro-1,1′,3, 3′-tetraethyl benzimidazole-carbocyanine iodide probe (JC-1; Molecular Probes), was also used to confirm data obtained by JC-1 as described [31].

#### Mitochondrial reactive oxygen species

Cells were incubated with 5 μM MitoSOX (red mitochondrial superoxide indicator, Thermo Fisher Scientific) in complete medium, for 30 min at 37 °C.

#### Cytoplasmic ROS

Cells were incubated with 1 μM of dihydroethidium (Molecular Probes) or 10 μM dihydrorhodamine 123 (Molecular Probes) for 15 min at 37 °C for superoxide anion and hydrogen peroxide detection, respectively.

#### GSH intracellular level

Monochlorobimane (MCB, Molecular Probes) was added to the cell suspension to a final concentration of 40 μM and the cells were maintained at room temperature in the dark for 20 min prior to analysis.

Acquisition of the samples was performed immediately after cell staining on a FACSCalibur flow cytometer (BD Biosciences, San Jose, CA, USA) equipped with a 488 argon laser and with a 635 red diode laser or by an LRS II cytometer (Becton Dickinson) equipped with a 488-Argon laser and a UVB laser (for GSH) and at least 10,000 events per sample were run. Data were analyzed using the Cell Quest Pro software (BD Biosciences) or the DIVA software (Becton Dickinson).

Alternatively, acquisition of the samples was performed by a multimode plate reader detecting luminescence, fluorescence and absorbance (Promega Inc.). Fluorescence values were normalized on the basis of protein concentration, as measured by absorbance using the same instruments.

### Intracranial implantation of GSCs into immunocompromised mice

NOD-SCID mice (male; 4–6 week old; Charles River, Italy) were implanted intracranially with 2 × 10^5^ GFP-expressing GSC#1 resuspended in 5 μl of serum-free medium. For brain grafting, the mice were anesthetized with intraperitoneal injection of diazepam (2 mg/100 g) followed by intramuscular injection of ketamine (4 mg/100 g). Animal skulls were immobilized in a stereotactic head frame and a burr hole was made 2 mm right of the midline and 1 mm anterior to the coronal suture, and cells were slowly injected using the tip of a 10-μl Hamilton microsyringe placed at a depth of 3 mm from the dura. One week after grafting, the mice were randomly assigned to four groups and treated according to the following protocol.

**Table:** 

Group	n	Treatment	Dose	Schedule
I	4	Saline 2 ml		Three times/week (Three Weeks)
II	4	Elesclomol	25 mg/kg	Three times/week (Three Weeks)
III	4	Temozolomide (TMZ)	50 mg/kg	Three times/week (Three Weeks)
IV	4	Temozolomide (TMZ) + Elesclomol	50 mg/kg + 25 mg/kg	Three times/week (Three Weeks)

During treatment, the body weight and neurological status were monitored daily. Eight weeks after grafting, the mice were deeply anesthetized and transcardially perfused with 0.1 M PBS (pH 7.4) followed by 4% paraformaldehyde in 0.1 M PBS. The brain was removed, stored in 30% sucrose buffer overnight at 4 °C, and serially cryotomed at 25 μm on the coronal plane. The liver, lung, kidney, and spleen of mice were also as assessed by conventional histology.

### Immunofluorescence analysis of tumors in brain slices

After perfusion, the brains were removed from the skull and post-fixed in the same fixative overnight at 4 °C. After rinsing in phosphate buffer, brains were cryoprotected by sequential incubation with sucrose solutions, 15 and 30% both overnight at 4 °C. Free-floating sections were then analysed for immunofluorescence [32], while they were kept for long storage at − 20 °C in freezing solution (containing ethylene glycol and glycerol). After rinsing in PBS, sections were incubated in 10% normal horse serum in phosphate buffer containing 0.2% Triton X-100 for 30 min to reduce non-specific binding. These sections were then incubated with primary antibodies (rabbit anti Ki67 1:2, Ventana Inc.; rabbit anti GFAP 1:500, Chemicon International, Temecula, CA, USA; mouse anti GFAP 1:500; Invitrogen, Carlsbad, CA, USA) diluted in phosphate buffer containing 2% normal horse serum and 0.2% Tritox X-100, overnight at 4 °C. After rinsing, the sections were incubated with labelled secondary antibodies (Alexa Fluor Molecular Probes) for 1 h at RT. After a thorough rinse, the sections were incubated in phosphate buffer containing a Hoechst for 10 min at RT; sections were mounted on slides and coverslipped with antifade medium (ProLong Glass mountant, Invitrogen). Images were obtained with a Laser Scanning Confocal Microscope (Olympus FluoView FV1000, Olympus Inc.). Brain sections for the different experimental groups (*n* = 6) were analysed for the presence and distribution GFP+ cells; large images were then acquired with an Eclipse 80i Nikon Fluorescence Microscope (Nikon Instruments, Amsterdam, Netherlands) and the entire brain slice was reconstructed by using Adobe Photoshop software. The cranio-caudal extension of the brain area invaded by fluorescent tumor cells was assessed on serial coronal sections. The volume of the brain invaded by the tumor was determined according to the equation, *V* = (*a*2 x *b*)/2, where *a* is the mean transverse diameter of the tumor calculated on coronal sections through the tumor epicenter and *b* is the cranio-caudal extension of the tumor [33].

Cell proliferation and astrocyte differentiation were evaluated by immunostaining with Ki67 (rabbit, Ventana Inc.) and GFAP (mouse, Invitrogen; rabbit Chemicon International), respectively. Images were obtained with a Laser Scanning Confocal Microscope (Flouview FV1000, Olympus Inc.). Cells positive for Ki67/field were counted using Fiji-ImageJ software.

### Statistical analysis

Results of fluorimetric and flow cytometry evaluations are presented as the mean ± standard deviation (SD). For tests of significance between groups, one-way analysis of variance (ANOVA) was performed. Comparisons between two groups or four groups were performed using the unpaired Student’s t test or two-way ANOVA with post hoc tests, respectively (Graphpad software, ver. 5.0). *P* < 0.05 was considered to indicate a statistically significant difference. All measurements were performed at least in three independent experiments.

Representation of compound library drug screening as well as unsupervised and statistical analysis of RPPA data and related figures were produced by means of R v4.0.2 [34], RStudio v1.3 [35] using the following libraries: tidyverse, readxl, NMF, FactoMineR, factoextra, ggpubr, grid, RColorBrewer, knitr, tcltk, openxlsx, data.table, plyr, car, coin, nortest, exactRankTests, sf, multcomp, Rfit, PMCMRplus, plotly, htmlwidgets. The derivation of unsupervised RPPA results is postponed to the Rmarkdown.

In order to perform binary statistical comparisons of RPPA data for all levels of cell growth conditions (GSC and GdEC) and time (6, 12, 24 and 36 h), we decided to i) pool values from cell lines and elesclomol concentrations (10, 100 and 1000 nM) and, since only part of the data presented a normal distribution, ii) use non-parametric statistics (Wilcoxon rank sum test). The resulting *p* values were adjusted following FDR correction.

Enrichment analyses were performed by taking advantage of g:Profiler [36], KEGG mapper [37] and WebGestalt [38].

## Results

### GSC-derived endothelial cells generate less differentiated and highly proliferating tumor xenografts

**Fig. 1:**
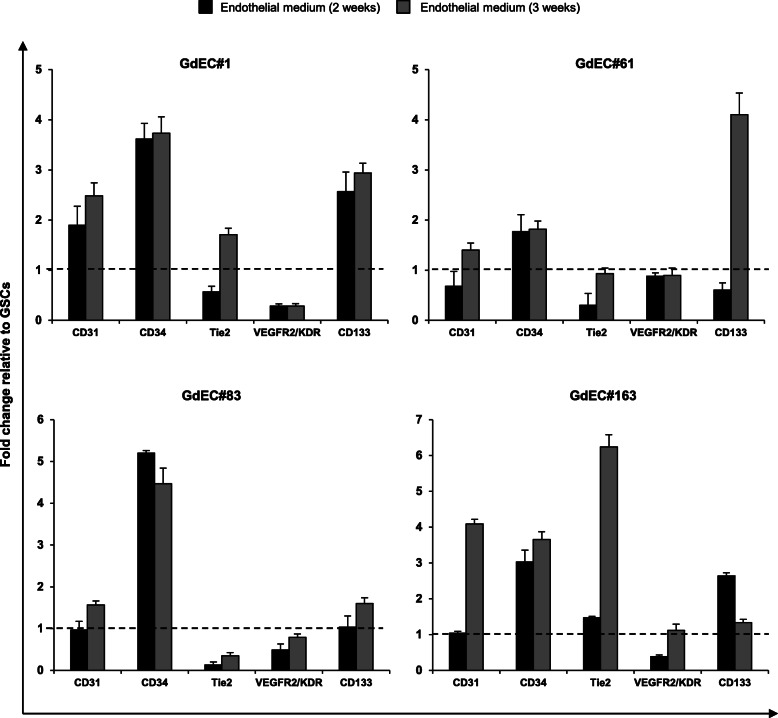
GSCs cultured under hypoxia in endothelial conditions upregulate endothelial markers. Cytofluorimetric evaluation of CD31, CD34, Tie2, VEGFR2/KDR and CD133 endothelial marker expression after two and three weeks of culture in endothelial conditions under hypoxia. Four GSC lines (i.e. GSC#1, #61, #83 and #163) isolated from different GBM patients are shown. The baseline (dashed line at value 1) represents the expression of the same markers in the four GSC lines cultivated in stem cell medium in normoxia conditions, used as reference. Results are reported as mean ± SD (*n* = 3)

Previously, we showed that GSCs cultivated under endothelial cell conditions develop morphological, phenotypical, and functional features of endothelial cells [22]. Moreover, GSC-derived endothelial cells are characterized by the expression of endothelial progenitor cell (EPC)-specific markers such as CD34 and CD133. The expression of endothelial markers changes dynamically during lineage development and it is affected by cell confluency [39]. To assess the tumorigenic potential of GdECs, four patient-derived GSC lines (i.e. GSC#1, #61, #83 and #163) (Supplementary Table 3) were cultured under hypoxia in endothelial cell conditions, i.e. stem cell medium with serum and endothelial growth factors. Differently from GSCs cultured under normoxia in stem cell medium, 2 weeks after seeding the GdECs from the four lines upregulated several endothelial markers though at different levels, confirming their ability to acquire an endothelial phenotype (Fig. 1 and Supplementary Fig. S1). Along with CD31, a cell marker of mature endothelium [40], we observed a consistent increase of CD34 that is widely regarded as a marker of vascular endothelial progenitor cells [41]. Then, to confirm the tumorigenic properties of GdECs and the role of CD34 in the tumorigenic process, we sorted CD34^−^/^low^ and CD34^high^ GdECs and grafted subcutaneously in athymic mice (Supplementary Fig. S2 A-C). Xenografts originated from CD34^−^/^low^ cells were small and showed features of differentiated tumors (Supplementary Fig. S2B). Conversely, those generated by CD34^high^ cells were more aggressive with areas of necrosis and high percentage of proliferating cells and showed an undifferentiated phenotype (Supplementary Fig. S2C). This experiment confirmed that the GdECs maintain their tumorigenic potential even after endothelial differentiation and that CD34 expression identifies in vivo the most aggressive tumor cells.

### The screening of a small molecule kinase inhibitor library identifies oxidative stress inducers as potential therapeutic tools

**Fig. 2:**
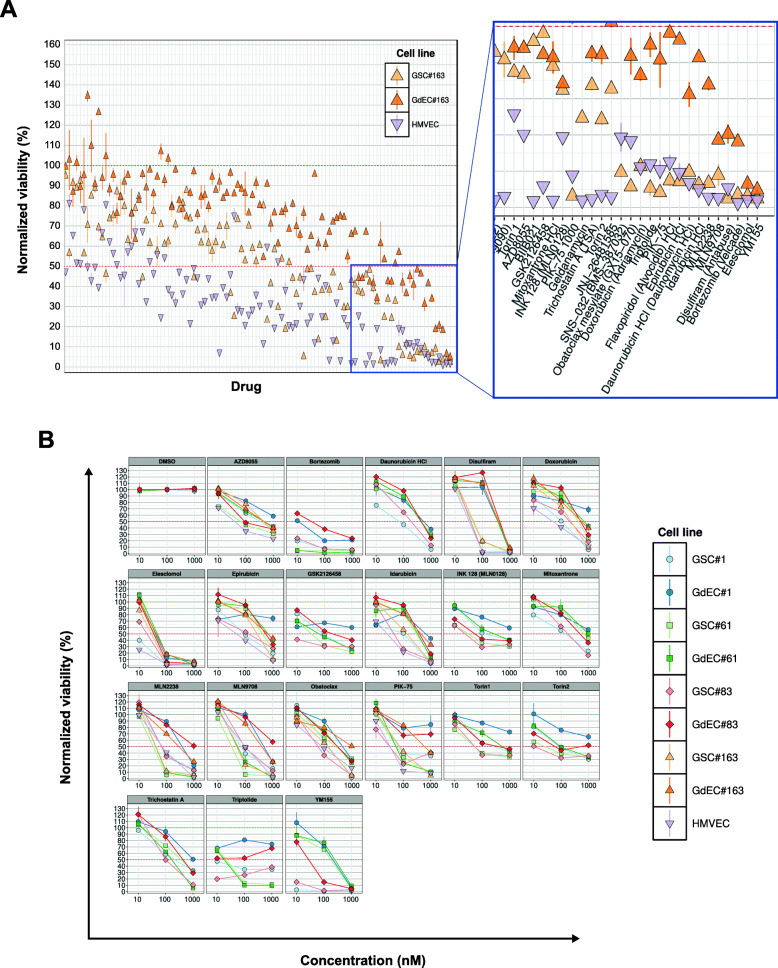
GSCs and GdECs show extraordinary resistance to targeted inhibition. **A** Kinase inhibitor library screening in a representative GSC line either in stem cell medium (GSC) or in endothelial conditions (GdEC) and in HMVECs. Cell viability is reported as mean ± SD (*n* = 3) of standardized values (*z score*) for each cell line treated with the kinase inhibitor library at 1 μM for 72 h. **B** Concentration-response assays on all the four selected GSC lines either in stem cell medium (GSC) or in endothelial conditions (GdEC), for the set of chemotherapeutics yielded a significant decrease in cell number. Cell viability is reported as mean ± SD (*n* = 3) for each cell line and drug tested for 72 h

Since the evidence that GSCs are able to transdifferentiate into GdECs, several studies have been focused on their role in tumor vascularization. Reportedly, the generation of GdECs is a hypoxia-dependent but VEGF-independent process, suggesting that GdECs are involved in the resistance to anti-VEGF therapy, and hence a potential target for GBM therapy [42]. For these reasons, we assessed the effect of a series of compounds able to counteract most of the GdEC survival pathways. A commercially available anti-cancer drug library was screened on one out of the four selected GSC lines, GSC#163, either cultivated in stem cell medium or transdifferentiated in GdECs. Such collection of bioactive compounds includes 349 experimental, investigational or FDA-approved kinase inhibitors targeting most cancer-related pathways (PI3K, HDAC, mTOR, MAPK, CDK, Aurora Kinase, JAK, etc.). Human dermal microvascular endothelial cells (HMVECs) were used as a control of normal (non-tumor) endothelial cells. After 72 h treatment, both GSCs and GdECs showed a low sensitivity to most of the compounds tested (Fig. 2A). However, a set of chemotherapeutics, as well as, inhibitors of Bcl-2 family, PI3K, HDAC, mTOR and 20S proteasome, yielded a significant decrease in the tumor cell number of GSCs and GdECs (Fig. 2A). Our functional data derived from in vitro kinase inhibition confirmed the existence of strong survival signals in both GSCs and GdECs that confer resistance to targeted inhibition. Since the screening was performed at a high concentration (i.e. 1 μM), to assess the specificity of kinase inhibitor effect and rule out off-target effects, we performed concentration-response assays on all the four selected GSC lines either in stem cell or endothelial cell culture conditions. Most of the compounds were inactive at submicromolar concentrations, as shown by markedly high EC50 values (half maximal effective concentration). Among the agents active at submicromolar concentrations, elesclomol (STA-4783), a potent oxidative stress inducer, was the most effective antiproliferative agent since both the GSCs grown in stem cell medium and those cultivated under endothelial medium showed a high degree of sensitivity (Fig. 2B). To evaluate the efficacy of elesclomol also in differentiated glioblastoma cell line, we performed a concentration-response assay on all the four selected GSC lines differentiated in glial conditions. The U87MG cell line was used as control of differentiated cell line. As shown in Supplementary Fig. S3, elesclomol exerts its antiproliferative effects also in U87MG and glial cells derived from differentiation of the four GSC lines used in this study.

### Elesclomol induced non-apoptotic copper-dependent cell death by promoting mitochondrial ROS production

**Fig. 3:**
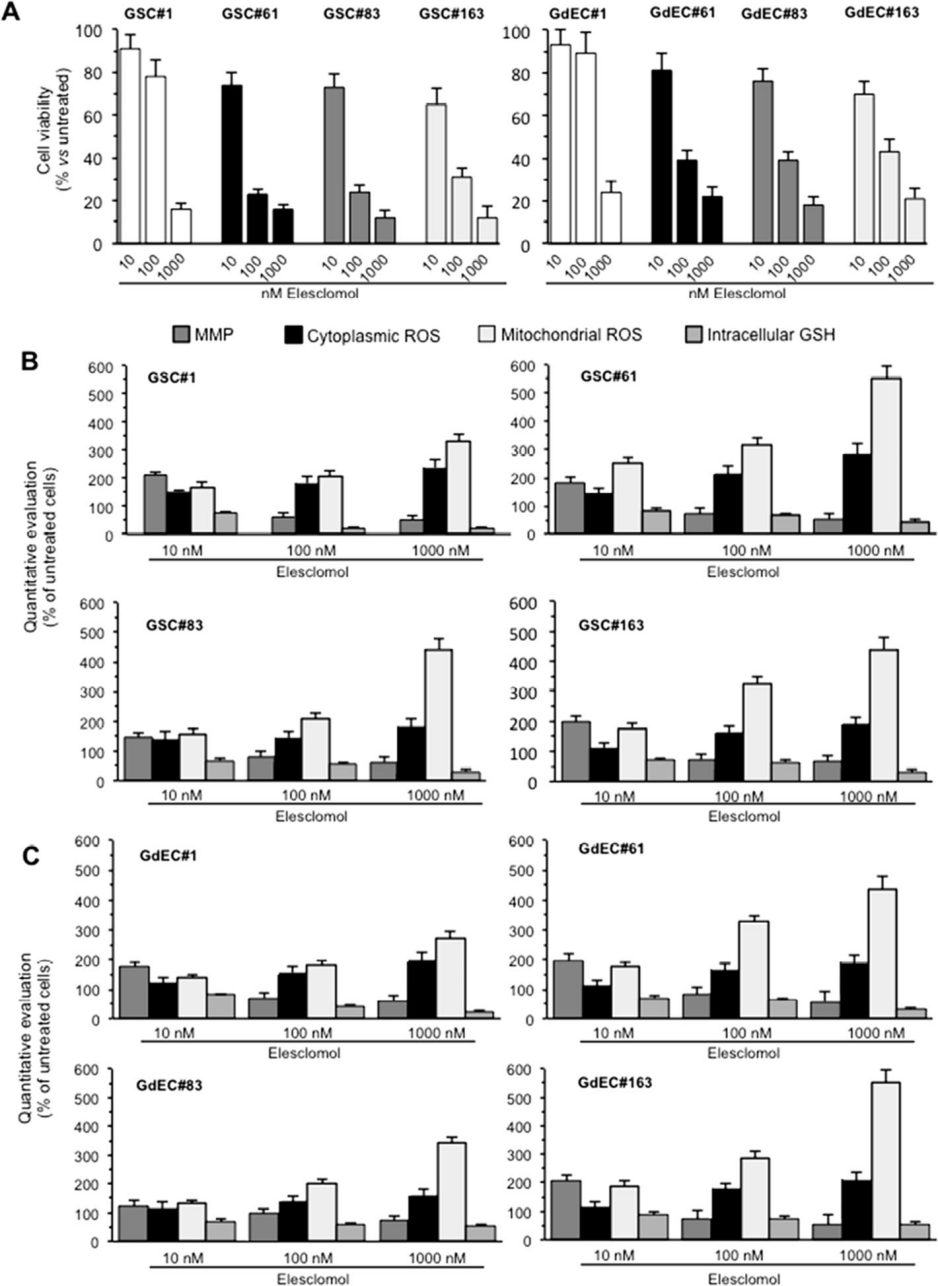
Elesclomol induced a dose dependent increase of cell death and mitochondrial ROS. **A** Fluorimetric evaluation of viability of four different GSC lines grown in stem cell (GSCs, *left panel*) or in endothelial medium (GdECs, *right panel*) after treatment with 10, 100 and 1000 nM elesclomol and staining with calcein-AM (which is retained in the cytoplasm of live cells). Numbers represent calcein-positive cells, calculated as percentage of control untreated cells. Fluorimetric evaluation of MMP (by TMRM dye), cytoplasmic ROS (by DHR123 dye), mitochondrial ROS (by MitoSox red dye), and GSH (by MCB dye) in **B** GSCs and **C** GdECs. Results obtained from three independent experiments performed in triplicate are reported as means ± SD

To go inside the mechanism of action of elesclomol, we analyzed a series of intracellular parameters, in the four GSC lines grown either in stem cell or in endothelial medium, by fluorimetric evaluations. As demonstrated by fluorimetric test performed in triplicate, 48 h treatment with elesclomol induced a dose dependent increase of cell death in either GSCs or GdECs (Fig. 3A). Cell treatment with elesclomol also induced mitochondrial membrane alterations, paralleled by a dramatic increase of mitochondrial ROS production, and a significant decrease in GSH levels, either in GSCs or GdECs (Fig. 3B and C, respectively), although the latter appeared slightly less sensitive to the drug. This agrees perfectly with what is reported by Modica-Napolitano and collaborators who identified the electron transport chain as a selective target of elesclomol [43]. Indeed, cytoplasmic ROS (i.e., by using also Hydroethidine and H_2_DCFDA, data not shown) increased significantly less markedly than mitochondrial ROS, thus indicating that mitochondrial stress may represent the *primum movens* of the elesclomol toxicity.

A more in-depth analysis of the mechanism of death was performed at the single cell level by flow cytometry using specific inhibitors for different types of cell death. Results obtained in GSCs treated with z-VAD (an apoptosis inhibitor), two antioxidants with different mechanism of action (e.g., N-acetylcysteine, NAC, or coenzyme Q10, CoQ), or specific inhibitors of necroptosis (necrostatin-1), ferroptosis (ferrostatin-1), and autophagy (3-methyladenine, 3-MA), indicated that none of the most common forms of death were responsible for the cytotoxic effects induced by elesclomol (Supplementary Fig. S4). Furthermore, neither CoQ nor NAC at the lowest concentration (5 mM) were able to significantly prevent the toxic effects induced by elesclomol. However, increasing the NAC concentration to 10 mM a significant, although not complete, protective effect was observed but only following treatment with elesclomol at the doses of 10 and 100 nM (Supplementary Fig. S4). It has been reported that elesclomol was able to chelate copper and transport it inside the mitochondria in cancer cells, where it induced an increase in oxygen radical production [44]. According with this, we found that the selective Cu^++^ chelator ammonium tetrathiomolybdate (TTM) was able to almost completely prevent the cytotoxic effect induced by elesclomol (Supplementary Fig. S5A). In addition, TTM also inhibited the dramatic increase (up to 400 times) of mitocondrial ROS production (Supplementary Fig. S5B) caused by the administration of the drug. At the mitochondrial membrane level, we observed a significant depolarization in cells treated with 100 and 1000 nM elesclomol while 10 nM of the drug induced an evident hyperpolarization of the mitochondrial membrane in cell lines GSC#1, #61 and #163, as revealed by cytofluorimetric analysis performed by using TMRM (Supplementary Fig. S5C) and JC-1 (data not shown) dyes. Cell treatment with TTM also prevented elesclomol-induced alterations of mitochondrial membrane potential (Supplementary Fig. S5C) and intracellular GSH reduction (Supplementary Fig. S5D). Moreover, HMVECs used as a control of normal (non-tumor) endothelial cell line, showed a trend that was completely overlapping with the tumor cell lines. In fact, even in these cells TTM was able to completely prevent the oxidative burst induced by the higher dose of elesclomol, consequently inhibiting cell death (Supplementary Fig. S6).

Our results demonstrated that elesclomol induced a unique form of copper-dependent cell death in GSCs, as it has already been described in other cellular models [45].

### Elesclomol impairs the ability of tumor cells to react to oxidative stress

We sought to use RPPA, an established high-throughput (phospho-) proteomic platform [46] to analyze GSCs and GdECs challenged with varying elesclomol concentrations and exposure time points.

**Fig. 4:**
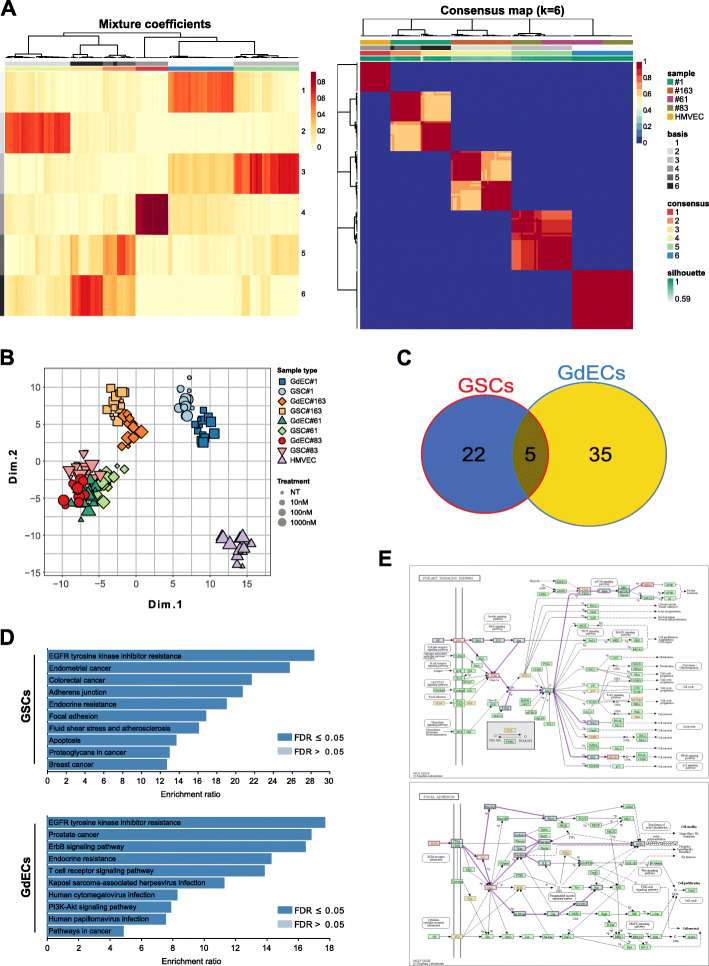
Reverse-Phase Protein microArrays (RPPA) analysis of HMVECs, GSCs and GdECs challenged with elesclomol. **A** NMF mixture coefficient and consensus map matrices heatmaps. The main clusters of samples correspond to individual cell lines, irrespective of growth conditions as well as of elesclomol concentration and time point. Color value ranges are reported at top-right of individual heatmaps. Color legends for annotation bars in both heatmaps are reported at the right of the panel. **B** PCA plot of dimensions 1 and 2. Sample and concentration scale legends are reported on the right of the plot. **C** Venn diagram of genes corresponding to significant antibodies emerging from statistical comparisons of vehicle and elesclomol samples, in GSCs grown in stem cell (GSCs) or endothelial cell (GdECs) conditions. The list of genes for each group (GSCs, GdECs, common) is available as Supplementary Table 4. **D** Histograms of the enrichment ratio of KEGG pathways relative to GSC- or GdEC-unique genes. Images were produced using the WEB-based GEne SeT AnaLysis Toolkit (WebGestalt, http://www.webgestalt.org/). **E** Representative PI3K-Akt Signaling and Focal Adhesion KEGG pathways showing mapped GdEC-unique genes and inferred network paths. Images were produced using the KEGG Mapper tool (Search Pathway, https://www.genome.jp/kegg/mapper.html)

Unsupervised data analysis performed via non-negative matrix factorization (NMF) revealed that the main differences in the levels of signaling-related endpoints are due to cell types (Fig. 4A, Supplementary Fig. S7A-B, and Supplementary Fig. S8). Principal Component Analysis (PCA) on data subsets according to cell lines, dosage and treatment time, showed that HMVECs substantially differ from GSCs in their signaling profile and, in line with the inherent molecular heterogeneity of GBM [47], RPPA expression patterns in GSCs were mainly dependent on their individual molecular scenario as well as on their growth culture conditions, i.e. standard stem cell or endothelial cell (Fig. 4B, Supplementary Fig. S9A-B, Supplementary Fig. S10A-B). Interestingly, when comparing vehicle- and elesclomol-treated cells, we found statistically significant differences in either GSC or GdEC, but not in HMVEC samples (Fig. 4C and Supplementary Table 4).

Finally, we performed gene enrichment analysis and found that genes corresponding to RPPA endpoints significantly deregulated by elesclomol are players in the PI3K-Akt, EGFR, focal adhesion, programmed cell death, oxidative stress and response to oxygen-containing compounds, as well as in integrin, axon guidance, and angiogenesis pathways (Fig. 4D-E). Overall, the RPPA analysis suggests that, by impairing the ability of cells to cope with oxidative stress, elesclomol ultimately interferes with GSCs’ survival and motility signals.

### Elesclomol increases sensitivity of GSCs and GdECs to TMZ

Based on the results that showed the efficacy of elesclomol on both GSCs and GdECs, we evaluated the ability of the latter to enhance the effects of TMZ. To this end, cytotoxicity assays were performed on the four cell lines previously used. The concentration of TMZ in the combination was chosen based on previous data [27]. Dose-response assays on all the four selected GSC lines were performed for setting the dose of elesclomol most suitable for the combination with TMZ (Supplementary Fig. S11).

**Fig. 5:**
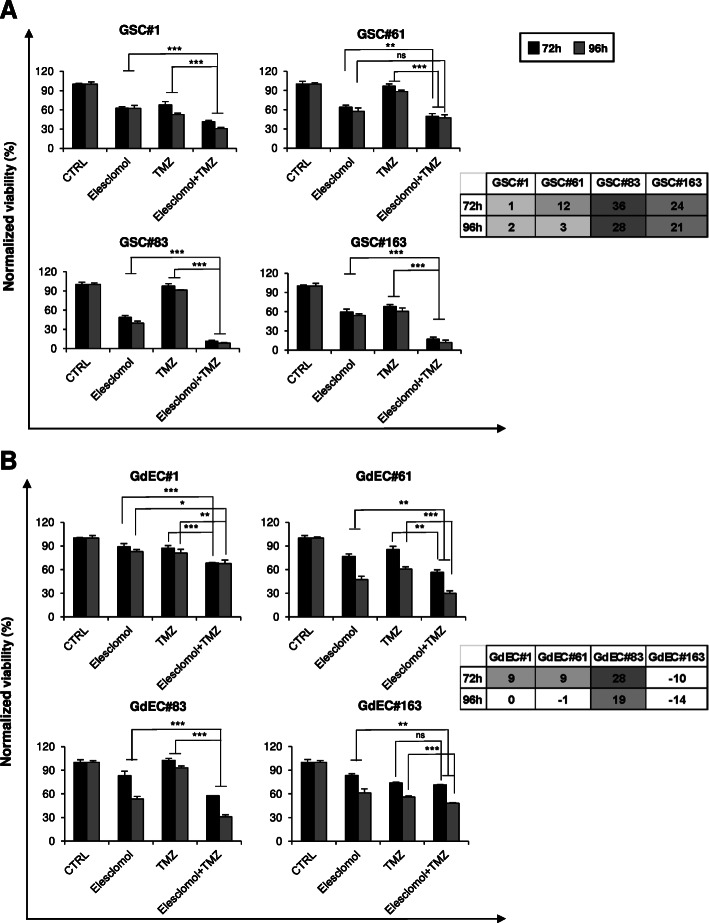
Elesclomol and TMZ combination is significantly more effective than TMZ alone. Cell viability assay performed on all the four selected GSC lines either in stem cell medium (**A**) or in endothelial conditions (**B**), treated with elesclomol alone, TMZ alone, and combined elesclomol and TMZ. Results obtained from three independent experiments are shown as percentage vs control untreated cells and reported as means ± SD. * *p* < 0.05; ** *p* < 0.01; *** *p* < 0.001 vs TMZ or eleclomol (Student-*t* test). The calculated excess over the predicted Bliss additivism model is shown in the tables on the right

Residual cellular viability following treatment showed that the combined treatment was significantly more effective than both TMZ and elesclomol alone in all the conditions tested except for combined treatment vs elesclomol alone in GSC#61 at 96 h of treatment (Fig. 5A).

To verify the efficacy of the combination also on the endothelial component of the tumor, the same assay was performed on the GdEC lines. Also in this case, combined treatment of the two compounds was significantly more effective than both TMZ and elesclomol alone in all the conditions tested except for combined treatment vs TMZ alone in GdEC#163 at 72 h of treatment (Fig. 5B), confirming the potential benefits of the addition of elesclomol. Moreover, excess over bliss analysis revealed that co-exposure to elesclomol and TMZ demonstrated different levels of synergy in the GSC and GdEC lines tested at both 72 h and 96 h of treatment (Fig. 5A-B).

### Elesclomol inhibits tumor growth and increases TMZ efficacy in vivo

**Fig. 6:**
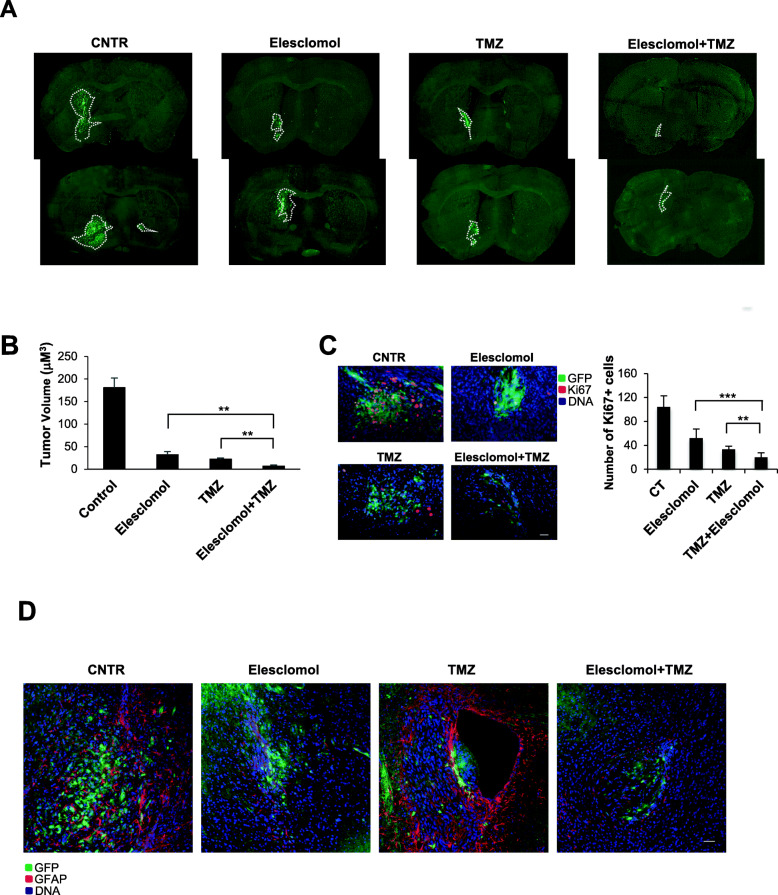
The in vivo effects of elesclomol on tumor xenografts in mice. **A** Coronal sections of mouse brain through the tumor implanted in the cerebral hemisphere. Representative images of coronal mouse brain sections at 8 weeks after the intracranial injection of GSCs. The white dashed lines indicate the intracranial tumors identified by the presence of GFP+ cells. Scale bar: 200 μm. **B** Graph showing the tumor volume for the intracranial tumors after 8 weeks from the injection of GSCs. ** *p* < 0.01 (Student-*t* test). **C** Representative images of brain tissue of mice stained using Hoechst and an antibody specific for Ki67. Scale bar: 20 μm. A reduction in the number of dividing cells is observed following the different antitumoral treatments with a particular significant effect for the combination elesclomol + TMZ, as indicated in the corresponding graph. ** *p* < 0.01; *** *p* < 0.001 (Student-*t* test). **D** Representative images of brain sections stained using Hoechst and an antibody specific for GFAP, marker for reactive astrocytes. Elesclomol alone or in combination with TMZ induces a reduction in the expression of GFAP in the cells around and within the tumoral area. Astrocytes appear faintly stained and with a thin morphology as compared with control and TMZ treatment alone brain samples. Scale bar: 20 μm

Intracerebral injection of GSC#1 into immunodeficient mice generates highly infiltrative tumor xenografts that closely mimic the behavior of malignant gliomas [22, 24]. This model was used to assess whether treatment with elesclomol may increase the antitumor effect of TMZ, as demonstrated by the in vitro assays. Stable green fluorescent protein (GFP)-expressing GSC#1 was grafted into the striatum of NOD-SCID mice. One week after grafting, the 4 mice per group were treated for 3 weeks either with saline, elesclomol alone, TMZ alone, or TMZ *plus* elesclomol. Analysis of tumor volumes at 8 weeks after grafting showed that mice treated with elesclomol alone, those treated with TMZ alone, and those treated with combined elesclomol and TMZ harbored significantly smaller tumor than saline treated mice (Fig. 6A). The combination of elesclomol *plus* TMZ was significantly more potent in inhibiting tumor growth than elesclomol alone (*p* = 0.0124) and TMZ alone (*p* = 0.0073) (Fig. 6B). These results suggest that elesclomol synergized with TMZ increasing its antitumor effect (excess over the predicted Bliss =1). It is worth to note that the control brain xenografts and those treated with TMZ alone showed tumor cells also in the hemisphere contralateral to the injection side, mainly along the cerebrospinal fluid (CSF) pathways. Such tumor cells, which were TMZ-resistant and capable to colonize the CSF paths, were not found in elesclomol-treated mice.

In addition, the combination of elesclomol *plus* TMZ significantly reduced tumor cell proliferation, as assessed by Ki67 labeling index, compared to both elesclomol alone and TMZ alone (*p* = 0.0003 and *p =* 0.002, respectively) (Fig. 6C). We next examined brain sections of the different experimental groups for the presence of reactive astrocytes using the expression of the glial fibrillary acidic protein (GFAP) marker. Figure 6D shows reactive astrocytes expressing high level of GFAP around and within the tumor mass.

Taken together, in vivo results show that elesclomol inhibits tumor growth and that the combination of elesclomol and TMZ enhances the antitumor effect of TMZ.

## Discussion

Conventional therapies lead to minimal and temporary benefits in patients with GBM. FDA has approved bevacizumab as a second line therapy but there is no evidence of efficacy on disease-related symptoms or survival. Conversely, it seems to help the tumor in its malign progression through the creation of hypoxic and nutrient-poor environment [20, 48]. The contribution of a subpopulation of tumor cells to vasculogenesis by transdifferentiating into endothelial-like cells may provide a mechanism to escape VEGF inhibition.

By screening more than 300 compounds we selected elesclomol to be effective on GSCs and on GdECs as well. Elesclomol is a small molecule anticancer agent that exhibits strong antitumor activity against a broad range of cancer cells including MDR (multi-drug resistance) cells [49]. Elesclomol exerts its anticancer activity via the induction of ROS in cancer cells, which results in apoptosis [50]. Biological data support the hypothesis that elesclomol generates ROS by chelating copper (II) and blocking redox cycling of copper (II) [44].

Elesclomol has demonstrated synergy with paclitaxel in preclinical models. A phase II study with elesclomol *plus* paclitaxel vs paclitaxel alone for stage IV metastatic melanoma was designed [51]. This study demonstrates encouraging efficacy and comparable toxicity for elesclomol *plus* paclitaxel compared to paclitaxel alone. However, a phase III trial comparing elesclomol *plus* paclitaxel versus paclitaxel alone in patients with stage IV melanoma failed the primary endpoint [52] and the drug was suggested to have potential use for other oncologic indications.

Elesclomol binds to copper Cu^++^ and deliver it into mitochondria, where it is released as Cu^+^ that can react with molecular oxygen generating ROS which, when produced in excess, can cause unmitigated oxidative stress and apoptotic death of cancer cells [44, 53]. Apoptosis also appears to be partly related with the ability of elesclomol and copper to inhibit the relaxation phase of topoisomerase I [54].

Recently it has been reported that elesclomol escorted copper to the mitochondria and increased cytochrome c oxidase levels in the brain [55].

Elesclomol is much more effective on tumor cells with high levels of ROS as melanoma cells, but it is non-toxic on normal cells as evidenced by tests on human keratinocytes [50] and in PBMCs [44].

Many evidences suggest that the tumor cells possess higher levels of intracellular ROS than normal cells [56, 57]. High levels of ROS in cancer are associated with promotion of cell proliferation, invasiveness and metastases [57–60]. It is well documented that the cancer stem cells (CSCs) contain lower levels of endogenous ROS compared to other non-CSCs tumor cells [61]. Low levels of intracellular ROS are considered a positive factor for the maintenance of quiescence and chemo/radio-resistance of CSCs [62], attributed to a greater expression of molecules with ROS scavenger function. Furthermore, it is reported that ROS are also involved in the differentiation of CSCs [61]. A study performed on GSCs has shown that GSCs within the tumor mass have low levels of cellular ROS although they are located in a hypoxic environment. The molecular mechanism is the up-regulation of peroxiredoxin 4 (PRDX4) in GSCs [63].

Our results suggest that, the oxidative stress is the main mechanism by which elesclomol acts on both GSCs and GdECs. In particular, cell treatment with elesclomol induced mitochondrial membrane alterations, increase of mitochondrial ROS production, and decrease in reduced GSH levels.

According to our data, ROS production has been observed in mitochondria with more than > 140 mV of membrane potential for many years already [64], as well as low GSH levels have been associated with hyperpolarization of the mitochondrial membrane [65]. In addition, GSH depletion, ROS generation, and increase of mitochondrial membrane potential have been reported to represent early molecular events of cell death [31]. The important role played by the drop in GSH levels in the cytotoxic effects induced by elesclomol would also be confirmed by the significant protective effect played by NAC when used at a sufficient concentration (10 mM).

Analysis of the mechanism of death revealed that, only avoiding drug activation into the mitochondria through a Cu^++^ chelating agent, such as TTM, the generation of large amounts of ROS responsible for cell death could be avoided. This observation confirms that elesclomol requires a redox active metal ion, mainly copper, for its functioning. Furthermore, investigating by RPPA the signaling pathways significantly deregulated by elesclomol we found genes that are key players in the PI3K-Akt, EGFR, focal adhesion, programmed cell death, oxidative stress and response to oxygen-containing compounds, as well as in integrin, axon guidance, and angiogenesis pathways. This suggests that, by impairing the ability of cells to cope with oxidative stress, elesclomol ultimately interferes with GSC survival and motility signals. Our in vivo findings support this hypothesis since the brain xenografts treated with elesclomol, both alone and combined with TMZ, did not show seeding of GSCs along the CSF pathways, which was frequently observed in control xenografts and in those treated with TMZ alone. The ability of the tumor cells to implant onto the ependymal layer and to grow simply relying on poor CSF nutrients are typical feature of GSCs that are highly motile in the brain and are able to survive in hostile environments [66].

Finally, our in vitro and in vivo data reveal that combined treatment with elesclomol and TMZ, is more effective than treatment with TMZ alone. This result suggests that targeting oxidative stress could represent a valuable strategy for novel therapies in GBM.

## Conclusions

The current standard therapy of GBM is widely applied however, life expectancy of GBM patients remains dismal. One hallmark of this tumor is the existence of hypoxic zones, which are enriched in GSCs. Hypoxia directly supports GSC self-renewal as well as controls stem cell plasticity. In such permissive microenvironment, GSCs are able to transdifferentiate into functional GSC-derived endothelial cells (GdECs). Searching for compounds able to interfere with cancer-related pathways in GBM, we found the oxidative stress inducer, elesclomol, as the most effective agent able to impair cell viability in both GSC and GdEC lines tested. The present study provides evidence that combined treatment with elesclomol and TMZ enhances the antitumor effect of TMZ alone both in vitro and in vivo, suggesting that targeting oxidative stress could represent a valuable strategy for novel therapies in GBM.

## Supplementary Information

**Additional file 1: Supplementary Table 1.** List of drugs used for small-molecule kinase inhibitor screening (10 mM in DMSO). **Supplementary Table 2.** List of antibodies used for Reverse-Phase Protein microArrays (RPPA) analysis. **Supplementary Table 3**. Patient and GSC line characteristics. **Supplementary Table 4.** List of genes corresponding to significant antibodies and grouped using the Venn diagram in Fig. 4C. **Supplementary Figure S1. A-D.** Morphological changes of the four GSC lines used in the study (**A**, GSC#1; **B**, GSC#61; **C,** GSC#83; **D**, GSC#163) after being induced to transdifferentiate for 2 weeks. *Left panel*, tumorspheres in stem cell medium; *right panel*, net-like structures under endothelial conditions (magnification 10X). **Supplementary Figure S2.** (**A**) Fluorescent-activated cell sorting dot plots of CD34^−/low^ and CD34^high^ GSC#163 after two weeks of culture in endothelial conditions under hypoxia. Percentage and squares indicate the sorted subpopulations of cells with different CD34-expression levels (*left*, IgG1 isotype control sample; *right,* CD34 sample). (**B-C**) Immunohistochemical analysis of CD34^low^ (B) and CD34^high^ (C) GdEC subcutaneous tumor xenografts based on the expression of the astrocytic marker glial fibrillary acidic protein (GFAP, *right panels*), showing tumors with different levels of differentiation. (*Left panels*, haematoxylin and eosin staining; magnification 200X). **Supplementary Figure S3.** Concentration-response assays on U87MG and all the four glial cell lines derived from the selected GSC lines. **Supplementary Figure S4.** Cytofluorimetric cell-by-cell analysis of viability in four different GSC lines treated with 10, 100, or 1000 nM elesclomol in the presence or absence of the following cell death inhibitors: z-VAD, necrostatin-1, ferrostatin-1, 3-MA, NAC, and CoQ at the indicated concentrations. Results obtained from four independent experiments are expressed as percentage vs control untreated cells and reported as means ± SD. **Supplementary Figure S5.** Cytofluorimetric cell-by-cell analysis of cell viability (**A**), mitochondrial ROS production (**B**), mitochondrial membrane potential (**C**), and GSH (**D**) in four different GSC lines treated with 10, 100, or 1000 nM elesclomol in the presence or absence of the copper chelating agent TTM. Results obtained from four independent experiments are expressed as percentage vs control untreated cells and reported as means ± SD. **Supplementary Figure S6.** Cytofluorimetric cell-by-cell analysis of cell viability, mitochondrial ROS production, mitochondrial membrane potential, and GSH in HMVECs, used as a control of nontumoral endothelial cell line, treated with 10, 100, or 1000 nM Elesclomol in the presence or absence of the copper chelating agent TTM. Results obtained from four independent experiments are expressed as percentage vs control untreated cells and reported as means ± SD. **Supplementary Figure S7.** Illustration of the rationale suitable for the choice of rank k, a critical parameter that defines the number of metagenes used to approximate the target matrix (Gaujoux & Seoighe, 2010). **A**) Measurements are applied to both real data (**circles**) and randomized data (**triangles**). The rationale for choosing rank stems on diverse metrics, **i**) trend of the cophenetic coefficient: Brunet et al. (2004) suggest choosing the smallest value of k for which there is a decrease in the trend of the cophenetic; **ii**) trend of the dispersion coefficient introduced by Kim & Park. (2007); **iii**) explained variance by increasing rank; **iv**) trend of residuals; **v**) trend of RSS: Hutchins et al. (2008) suggest taking the first rank value for which we have an inflection point. Frigyesi et al. (2008) instead consider the first rank value for which the decrease of the RSS on real data is less than the decrease of the RSS on the random data; **vi**) silhouette values measured on the matrices of the base, of the coefficients and the consensus matrix; **vii**) trend of the sparseness introduced by Hoyer (2004). **B**) Multiple consensus maps corresponding to different value of k. **Supplementary Figure S8.** Heatmap of the most important antibodies in each of the **k = 6** metagenes resulting from the model. **Supplementary Figure S9.** Principal Component Analysis (PCA) algorithm applied to Elesclomol data, whereby each cell line is considered as a function of the antibodies. **A**) Scree plot. Given the low amount of variance explained by the variables above the fifth, we considered up to 5 principal components. **B**) Biplots using cell lines and growth conditions as scores. Ellipses represent the 95% probability of finding sample score values. **Supplementary Figure S10.** Principal Component Analysis (PCA) biplots of components of the antibodies using (**A**) Time and (**B**) treatment, respectively. Ellipses represent the 95% probability of finding sample score values. **Supplementary Figure S11.** Concent ration-response assays on all the four selected GSC lines for setting the dose of Elesclomol most suitable for the combination with TMZ.

## Data Availability

The data produced and analyzed are available from the corresponding author on reasonable request.

## Abbreviations

GBM: Glioblastoma
GSCs: Glioblastoma stem-like cells
GdECs: GSC-derived endothelial cells
ROS: Reactive oxygen species
TMZ: Temozolomide
VEGF-A: Vascular endothelial growth factor A
HIF-1: Hypoxia-inducible factor-
OS: Overall survival
PLXDC1: Plexin Domain Containing 1
RPPA: Reverse-Phase Protein Arrays
EGF: Epidermal growth factor
b-FGF: Basic fibroblast growth factor
STR: Short Tandem Repeat
HMVECs: Human microvascular endothelial cells
FFPE: Formalin-fixed, paraffin-embedded
TTM: Ammonium tetrathiomolybdate
NMF: Non-negative matrix factorization
PCA: Principal Component Analysis
CSCs: Cancer stem cells
PRDX4: Peroxiredoxin 4
