# Supplementary material for: Elesclomol-induced increase of mitochondrial reactive oxygen species impairs glioblastoma stem-like cell survival and tumor growth
Source: J Exp Clin Cancer Res. 2021 Jul 12;40:228. doi: 10.1186/s13046-021-02031-4 (PMC8273992; doi:10.1186/s13046-021-02031-4)
Supplement: Supplementary file 1 — Additional file 1: Supplementary Table 1. List of drugs used for small-molecule kinase inhibitor screening (10 mM in DMSO). Supplementary Table 2. List of antibodies used for Reverse-Phase Protein microArrays (RPPA) analysis. Supplementary Table 3. Patient and GSC line characteristics. Supplementary Table 4. List of genes corresponding to significant antibodies and grouped using the Venn diagram in Fig. 4C. Supplementary Figure S1. A-D. Morphological changes of the four GSC lines used in the study (A, GSC#1; B, GSC#61; C, GSC#83; D, GSC#163) after being induced to transdifferentiate for 2 weeks. Left panel, tumorspheres in stem cell medium; right panel, net-like structures under endothelial conditions (magnification 10X). Supplementary Figure S2. (A) Fluorescent-activated cell sorting dot plots of CD34−/low and CD34high GSC#163 after two weeks of culture in endothelial conditions under hypoxia. Percentage and squares indicate the sorted subpopulations of cells with different CD34-expression levels (left, IgG1 isotype control sample; right, CD34 sample). (B-C) Immunohistochemical analysis of CD34low (B) and CD34high (C) GdEC subcutaneous tumor xenografts based on the expression of the astrocytic marker glial fibrillary acidic protein (GFAP, right panels), showing tumors with different levels of differentiation. (Left panels, haematoxylin and eosin staining; magnification 200X). Supplementary Figure S3. Concentration-response assays on U87MG and all the four glial cell lines derived from the selected GSC lines. Supplementary Figure S4. Cytofluorimetric cell-by-cell analysis of viability in four different GSC lines treated with 10, 100, or 1000 nM elesclomol in the presence or absence of the following cell death inhibitors: z-VAD, necrostatin-1, ferrostatin-1, 3-MA, NAC, and CoQ at the indicated concentrations. Results obtained from four independent experiments are expressed as percentage vs control untreated cells and reported as means ± SD. Supplementary Figure S5. C [file 13046_2021_2031_MOESM1_ESM.pdf]

Supplementary Table 1. List of drugs used for small-molecule kinase inhibitor screening (10mM in DMSO).

CATALOG #	CAS #	COMPOUND NAME OR ID NUMBER	M.W.	TARGET
S2250	989-51-5	(-)-Epigallocatechin gallate	458,37	DNA damage/DNA repair
S1141	75747-14-7	17-AAG (Tanespimycin)	585,69	HSP (e.g. HSP90)
S1142	467214-21-7	17-DMAG HCl (Alvespimycin)	653,21	HSP (e.g. HSP90)
S1233	362-07-2	2-Methoxyestradiol	302,41	HIF
S2767	5142-23-4	3-Methyladenine	149,15	Autophagy
S2697	844499-71-4	A-769662	360,39	AMPK
S1123	154229-19-3	Abiraterone (CB-7598)	349,51	P450 (e.g. CYP17)
S1210	59-05-2	Abitrexate (Methotrexate)	454,44	DHFR
S1001	923564-51-6	ABT-263 (Navitoclax)	974,61	Bcl-2
S1002	852808-04-9	ABT-737	813,43	Autophagy
S1165	141430-65-1	ABT-751	371,41	Microtubule Associated
S1004	912444-00-9	ABT-888 (Veliparib)	244,29	PARP
S1209	51-21-8	Adrucil (Fluorouracil)	130,08	DNA/RNA Synthesis
S1486	497839-62-0	AEE788 (NVP-AEE788)	440,58	EGFR
S1011	439081-18-2	Afatinib (BIBW2992)	485,94	EGFR
S2178	328543-09-5	AG14361	320,39	PARP
S1278	645-05-6	Altretamine (Hexalen)	210,28	DNA damage/DNA repair
S2719	945595-80-2	AMG 900	503,58	Aurora Kinase
S1672	125-84-8	Aminoglutethimide (Cytadren)	232,28	Aromatase
S1244	850879-09-3	Amuvatinib (MP-470)	447,51	c-Kit
S3172	58579-51-4	Anagrelide HCl	292,55	PDE
S1188	120511-73-1	Anastrozole	293,37	Aromatase
S1140	401900-40-1	Andarine (GTX-007)	441,36	Androgen Receptor
S2799	658084-64-1	APO866 (FK866)	391,51	NAMPT
S1189	170729-80-3	Aprepitant (MK-0869)	534,43	Substance P
S2244	935881-37-1	AR-42 (HDAC-42)	312,36	HDAC
S2754	1071992-99-8	AT-406	561,71	E3 Ligase
S1524	844442-38-2	AT7519	382,24	CDK
S1134	896466-04-9	AT9283	381,43	Aurora Kinase
S1069	747412-49-3	AUY922 (NVP-AUY922)	465,54	HSP (e.g. HSP90)
S1005	319460-85-0	Axitinib	386,47	c-Kit
S2731	1124329-14-1	AZ 3146	452,55	Kinesin
S2746	878739-06-1	AZ628	451,52	Raf
S1782	320-67-2	Azacitidine (Vidaza)	244,20	DNA Methyltransferase
S1721	446-86-6	Azathioprine (Azasan, Imuran)	277,26	Rho
S1008	606143-52-6	AZD6244 (Selumetinib)	457,68	MEK
S1532	860352-01-8	AZD7762	362,42	Chk
S1555	1009298-09-2	AZD8055	465,54	mTOR
S1147	722544-51-6	Barasertib (AZD1152-HQPA)	507,56	Aurora Kinase
S2913	19542-67-7	BAY 11-7082 (BAY 11-7821)	207,25	E2 conjugating
S1085	414864-00-9	Belinostat (PXD101)	318,35	HDAC
S1212	3543-75-7	Bendamustine HCL	394,72	DNA damage/DNA repair
S1689	1247-42-3	Betapar (Meprednisone)	372,46	n.a.

S2098	153559-49-0	Bexarotene	348,00	n.a.
S1009	915019-65-7	BEZ235 (NVP-BEZ235)	469,55	ATM/ATR
S1109	755038-02-9	BI 2536	521,66	PLK
S2235	755038-65-4	BI6727 (Volasertib)	618,81	PLK
S1010	656247-17-5	BIBF1120 (Vargatef)	539,62	FGFR
S1186	321674-73-1	BIBR 1532	331,36	Telomerase
S1190	90357-06-5	Bicalutamide (Casodex)	430,37	Androgen Receptor
S1175	848695-25-0	BIIB021	318,76	HSP (e.g. HSP90)
S1574	285983-48-4	BIRB 796 (Doramapimod)	527,66	p38 MAPK
S2247	944396-07-0	BKM120 (NVP-BKM120)	410,39	PI3K
S1214	9041-93-4	Bleomycin sulfate	1512,6<br>2	DNA/RNA Synthesis
S1561	1025720-94-8	BMS 777607	512,89	Axl
S2201	1174046-72-0	BMS 794833	468,84	c-Met
S1056	714971-09-2	BMS-599626 (AC480)	567,01	EGFR
S1013	179324-69-7	Bortezomib (Velcade)	384,24	Proteasome
S1014	380843-75-4	Bosutinib (SKI-606)	530,45	Src
S1084	649735-46-6	Brivanib (BMS-540215)	370,38	FGFR
S1097	957217-65-1	BTZ043 racemate	431,39	n.a.
S1692	55-98-1	Busulfan (Myleran, Busulfex)	246,30	DNA damage/DNA repair
S1274	702675-74-9	BX-795	591,47	IκB/IKK
S2226	870281-82-6	CAL-101 (GS-1101)	415,42	PI3K
S2760	842133-18-0	Canagliflozin	444,52	SGLT
S1156	154361-50-9	Capecitabine (Xeloda)	359,35	DNA/RNA Synthesis
S1215	41575-94-4	Carboplatin	371,25	DNA/RNA Synthesis
S1289	61422-45-5	Carmofur	257,26	DNA/RNA Synthesis
S1017	288383-20-0	Cediranib (AZD2171)	450,51	VEGFR
S1261	169590-42-5	Celecoxib	381,37	COX
S2806	1257704-57-6	CEP33779	462,57	JAK
S2699	1007207-67-1	CH5132799	377,42	mTOR
S2924	252917-06-9<br>(free base)	CHIR-99021 (CT99021) HCl	501,80	GSK-3
S2406	481-74-3	Chrysophanic acid (Chrysophanol)	254,24	EGFR
S1020	212631-79-3	CI-1040 (PD184352)	478,67	MEK
S1166	15663-27-1	Cisplatin	300,05	DNA/RNA Synthesis
S1199	4291-63-8	Cladribine	285,69	DNA/RNA Synthesis
S1217	50-18-0	Clafen (Cyclophosphamide)	261,09	DNA/RNA Synthesis
S1218	123318-82-1	Clofarabine	303,68	DNA/RNA Synthesis
S2398	303-98-0	Coenzyme Q10 (CoQ10)	863,34	n.a.
S2245	1080622-86-1	CP-466722	349,35	ATM/ATR
S2730	670220-88-9	Crenolanib (CP-868596)	443,54	PDGFR
S1068	877399-52-5	Crizotinib (PF-02341066)	450,34	ALK
S1194	1012054-59-9	CUDC-101	434,49	EGFR
S2248	1009820-21-6	CX-4945 (Silmitasertib)	349,77	PKC
S1171	693228-63-6	CYC116	368,46	Aurora Kinase
S1146	4449-51-8	Cyclopamine	411,62	Hedgehog
S2057	6055-19-2	Cyclophosphamide monohydrate	279,10	DNA damage/DNA repair

S2286	59865-13-3	Cyclosporin A (Cyclosporine A)	1202,6<br>1	Calcineurin
S2219	1056634-68-4	Cyt387	414,46	JAK
S1648	147-94-4	Cytarabine	243,22	DNA/RNA Synthesis
S1221	4342-03-4	Dacarbazine (DTIC-Dome)	182,18	DNA/RNA Synthesis
S2727	1110813-31-4	Dacomitinib (PF299804,PF-00299804)	469,94	EGFR
S2772	211513-37-0	Dalcetrapib (JTT-705)	389,59	CETP
S1107	827318-97-8	Danusertib (PHA-739358)	474,55	Aurora Kinase
S1548	461432-26-8	Dapagliflozin	408,87	SGLT
S2215	208255-80-5	DAPT (GSI-IX)	432,46	Beta Amyloid
S1021	302962-49-8	Dasatinib (BMS-354825)	488,01	Bcr-Abl
S3035	23541-50-6	Daunorubicin HCl (Daunomycin HCl)	563,98	Telomerase
S2634	1020172-07-9	DCC-2036 (Rebastinib)	553,59	Bcr-Abl
S1200	2353-33-5	Decitabine	228,21	DNA Methyltransferase
S1022	572924-54-0	Deforolimus (Ridaforolimus)	990,21	mTOR
S2826	183321-86-0	Desmethyl Erlotinib (CP-473420)	379,41	EGFR
S1322	50-02-2	Dexamethasone	392,46	Autophagy
S3124	1177-87-3	Dexamethasone acetate	434,50	Interleukin receptor
S1893	5959-95-5	D-glutamine	146,14	n.a.
S1201	16208-51-8	Dimesna	326,34	n.a.
S1680	97-77-8	Disulfiram (Antabuse)	296,54	ALDH
S1537	117570-53-3	DMXAA (ASA404)	282,29	VDA
S1148	114977-28-5	Docetaxel (Taxotere)	807,88	Microtubule Associated
S1375	130693-82-2	Dorzolamide HCl	360,90	Carbonic Anhydrase
S1018	405169-16-6	Dovitinib (TKI-258)	392,43	c-Kit
S1467	54573-75-0	Doxercalciferol (Hectorol)	412,65	n.a.
S1208	25316-40-9	Doxorubicin (Adriamycin)	579,98	Autophagy
S1164	417716-92-8	E7080 (Lenvatinib)	426,85	VEGFR
S1052	488832-69-5	Elesclomol	400,50	HSP (e.g. HSP90)
S1181	934353-76-1	ENMD-2076	375,47	Aurora Kinase
S1053	209783-80-2	Entinostat (MS-275, SNDX-275)	376,41	HDAC
S1055	170364-57-5	Enzastaurin (LY317615)	515,61	PKC
S1223	56390-09-1	Epirubicin HCl	579,98	Topoisomerase
S1297	152044-53-6	Epothilone A	493,66	Microtubule Associated
S1364	152044-54-7	Epothilone B (EPO906)	507,68	Microtubule Associated
S1023	183319-69-9	Erlotinib HCl	429,90	Autophagy
S1709	50-28-2	Estradiol	272,38	Estrogen/progestogen Receptor
S1665	53-16-7	Estrone	270,37	Estrogen/progestogen Receptor
S1225	33419-42-0	Etoposide (VP-16)	588,56	Topoisomerase
S1120	159351-69-6	Everolimus (RAD001)	958,22	mTOR
S1227	82640-04-8	Evista (Raloxifene HCl)	510,04	Estrogen/progestogen Receptor
S1541	49843-98-3	EX 527	248,71	Sirtuin
S1196	107868-30-4	Exemestane	296,40	Aromatase
S1655	163222-33-1	Ezetimibe (Zetia)	409,40	n.a.
S1547	144060-53-7	Febuxostat (Uloric)	316,37	Xanthine oxidase
S5002	162359-56-0	Fingolimod (FTY720)	343,90	Bcr-Abl

S2679	131740-09-5	Flavopiridol (Alvocidib) HCl	438,30	CDK
S1299	50-91-9	Floxuridine (Fludara)	246,19	DNA/RNA Synthesis
S1491	21679-14-1	Fludarabine (Fludara)	285,23	DNA/RNA Synthesis
S1229	75607-67-9	Fludarabine Phosphate (Fludara)	365,21	DNA/RNA Synthesis
S1908	13311-84-7	Flutamide (Eulexin)	276,21	P450 (e.g. CYP17)
S1909	93957-55-2	Fluvastatin sodium (Lescol)	433,45	HMG-CoA Reductase
S2208	566-48-3	Formestane	302,41	Aromatase
S1300	17902-23-7	Ftorafur	200,17	DNA/RNA Synthesis
S1191	129453-61-8	Fulvestrant (Faslodex)	606,77	Estrogen/progestogen Receptor
S1159	888216-25-9	Ganetespib (STA-9090)	364,40	HSP (e.g. HSP90)
S1104	905281-76-7	GDC-0879	334,37	Raf
S1065	957054-30-7	GDC-0941	513,64	PI3K
S1025	184475-35-2	Gefitinib (Iressa)	446,90	EGFR
S2713	30562-34-6	Geldanamycin	560,64	Autophagy
S1714	95058-81-4	Gemcitabine (Gemzar)	263,20	Autophagy
S1149	122111-03-9	Gemcitabine HCl (Gemzar)	299,66	Autophagy
S2303	303-45-7	Gossypol	518,56	Dehydrogenase
S2673	871700-17-3	GSK1120212 (Trametinib)	615,39	MEK
S1093	1089283-49-7	GSK1904529A	851,96	IGF-1R
S2658	1086062-66-9	GSK2126458	505,50	mTOR
S2193	929095-18-1	GSK461364	543,60	PLK
S1113	937174-76-0	GSK690693	425,48	Akt
S2630	405911-17-3	GW3965 HCl	618,51	Liver X Receptor
S2782	278779-30-9	GW4064	542,84	FXR
S1696	50-23-7	Hydrocortisone (Cortisol)	362,46	Glucocorticoid receptor
S1896	127-07-1	Hydroxyurea (Cytodrox)	76,05	DNA Synthesis
S1268	371242-69-2	IC-87114	397,43	PI3K
S1228	57852-57-0	Idarubicin HCl	533,95	Topoisomerase
S1302	3778-73-2	Ifosfamide	261,09	DNA/RNA Synthesis
S2475	152459-95-5	Imatinib (Gleevec)	493,60	PDGFR
S1026	220127-57-1	Imatinib Mesylate	589,71	Bcr-Abl
S1211	99011-02-6	Imiquimod	240,30	n.a.
S1087	160003-66-7	Iniparib (BSI-201)	292,03	PARP
S2811	1224844-38-5	INK 128 (MLN0128)	309,33	mTOR
S1198	97682-44-5	Irinotecan	586,68	Topoisomerase
S2217	136572-09-3	Irinotecan HCl Trihydrate (Campto)	677,18	Topoisomerase
S1379	4759-48-2	Isotretinoin	300,44	Hydroxylase
S1452	336113-53-2	Ispinesib (SB-715992)	517,06	Kinesin
S2476	84625-61-6	Itraconazole (Sporanox)	705,65	Hedgehog/Autophagy
S1172	881202-45-5	JNJ 26854165 (Serdemetan)	328,41	E3 Ligase
S1096	875320-29-9	JNJ-26481585	394,48	HDAC
S1114	943540-75-8	JNJ-38877605	377,35	c-Met
S1249	443797-96-4	JNJ-7706621	394,36	Aurora Kinase
S1226	938440-64-3	Ku-0063794	465,54	mTOR
S1092	587871-26-9	KU-55933	395,49	ATM/ATR
S1570	925701-49-1	KU-60019	547,67	ATM/ATR

S2700	897016-82-9	KX2-391	431,53	Src
S2111	231277-92-2	Lapatinib	581,06	EGFR
S1028	388082-77-7	Lapatinib Ditosylate (Tykerb)	925,46	EGFR
S2151	956697-53-3	LDE225 (NVP-LDE225, Erismodegib)	485,50	Smoothened
S2618	1062368-24-4	LDN193189	406,48	TGF-beta/Smad
S1029	191732-72-6	Lenalidomide (Revlimid)	259,26	TNF-alpha
S1235	112809-51-5	Letrozole	285,30	Aromatase
S1236	6035-45-6	Leucovorin Calcium	601,58	n.a.
S1003	796967-16-3	Linifanib (ABT-869)	375,41	CSF-1R
S1091	867160-71-2	Linsitinib (OSI-906)	421,49	IGF-1R
S1840	13010-47-4	Lomustine (CeeNU)	233,70	DNA Synthesis
S2610	50264-69-2	Lonidamine	321,16	Hexokinase
S2704	700874-71-1	LY2109761	441,52	TGF-beta/Smad
S2230	700874-72-2	LY2157299	369,42	TGF-beta/Smad
S1494	862507-23-1	LY2228820	612,74	p38 MAPK
S2626	911222-45-2	LY2603618 (IC-83)	436,30	Chk
S1105	154447-36-6	LY294002	307,34	Autophagy
S2003	376348-65-1	Maraviroc	513,67	CCR5
S1064	790299-79-5	Masitinib (AB1010)	498,64	c-Kit
S1250	915087-33-1	MDV3100 (Enzalutamide)	464,44	Androgen Receptor
S2567	71-58-9	Medroxyprogesterone acetate	386,52	Estrogen/progestogen Receptor
S1304	595-33-5	Megestrol Acetate	384,51	Androgen Receptor
S1305	50-44-2	Mercaptopurine	152,18	DNA/RNA Synthesis
S1735	19767-45-4	Mesna (Uromitexan, Mesnex)	164,18	n.a.
S2606	84371-65-3	Mifepristone (Mifeprex)	429,59	Estrogen/progestogen Receptor
S2485	70476-82-3	Mitoxantrone HCl	517,40	DNA damage/DNA repair
S2660	471905-41-6	MK-0752	442,90	Beta Amyloid
S1525	955365-80-7	MK-1775	500,60	Wee1
S1078	1032350-13-2	MK-2206 2HCl	480,39	Akt
S2180	1072833-77-2	MLN2238	361,03	Proteasome
S1133	1028486-01-2	MLN8237 (Alisertib)	518,92	Aurora Kinase
S2181	1201902-80-8	MLN9708	517,12	Proteasome
S1122	726169-73-9	Mocetinostat (MGCD0103)	396,44	HDAC
S1032	857876-30-3	Motesanib Diphosphate (AMG-706)	569,44	c-Kit
S1501	128794-94-5	Mycophenolate mofetil (CellCept)	433,49	Dehydrogenase
S2487	24280-93-1	Mycophenolic (Mycophenolate)	320,34	Dehydrogenase
S1213	121032-29-9	Nelarabine (Arranon)	297,27	DNA/RNA Synthesis
S2150	698387-09-6	Neratinib (HKI-272)	557,04	EGFR
S1033	641571-10-0	Nilotinib (AMN-107)	529,52	Bcr-Abl
S2775	31430-18-9	Nocodazole	301,32	Autophagy
S2638	503468-95-9	NU7441 (KU-57788)	413,49	DNA-PK
S1061	890090-75-2	Nutlin-3	581,50	E3 Ligase
S2686	1092499-93-8 (free base)	NVP-BSK805 2HCl	563,47	JAK
S1057	803712-79-0	Obatoclax mesylate (GX15-070)	413,49	Autophagy
S1060	763113-22-0	Olaparib (AZD2281)	434,46	PARP

S2205	183320-51-6	OSI-420	415,87	EGFR
S1220	728033-96-3	OSI-930	443,44	c-Kit
S1174	841205-47-8	Ostarine (MK-2866)	389,33	Androgen Receptor
S1224	61825-94-3	Oxaliplatin (Eloxatin)	397,29	DNA/RNA Synthesis
S2738	315183-21-2	PAC-1	392,49	Caspase
S1150	33069-62-4	Paclitaxel (Taxol)	853,91	Autophagy
S2238	914913-88-5	Palomid 529	406,43	mTOR
S1311	57248-88-1	Pamidronate Disodium	279,03	n.a.
S3012	444731-52-6	Pazopanib	437,52	c-Kit
S1090	783355-60-2	PCI-24781	397,42	HDAC
S2680	936563-96-1	PCI-32765 (Ibrutinib)	440,50	BTK
S1116	827022-32-2	PD 0332991 (Palbociclib) HCl	483,99	CDK
S1036	391210-10-9	PD0325901	482,19	MEK
S1079	183322-45-4	PD153035 HCl	396,67	EGFR
S1264	219580-11-7	PD173074	523,67	FGFR
S1392	257933-82-7	Pelitinib (EKB-569)	467,92	EGFR
S1135	150399-23-8	Pemetrexed (Alimta)	471,37	DHFR
S2013	869288-64-2	PF 573228	491,49	FAK
S2725	942487-16-3	PF-03814735	474,48	Aurora Kinase
S1094	956905-27-4	PF-04217903	372,38	c-Met
S2666	1196109-52-0	PF-3845	456,46	FAAH
S2890	717907-75-0	PF-562271	507,49	FAK
S2726	586379-66-0	PH-797804	477,30	p38 MAPK
S1070	477575-56-7	PHA-665752	641,61	c-Met
S1487	718630-59-2	PHA-793887	361,48	CDK
S2342	60-82-2	Phloretin (Dihydronaringenin)	274,27	SGLT
S1038	371935-74-9	PI-103	348,36	Autophagy
S1205	372196-77-5	PIK-75	488,74	DNA-PK
S1187	677338-12-4	PIK-90	351,36	PI3K
S1489	593960-11-3	PIK-93	389,88	PI3K
S2590	111025-46-8	Pioglitazone (Actos)	356,44	PPAR
S1567	19171-19-8	Pomalidomide	273,24	TNF-alpha
S1490	943319-70-8	Ponatinib (AP24534)	532,56	Bcr-Abl
S1622	53-03-2	Prednisone (Adasone)	358,43	Glucocorticoid receptor
S1995	366-70-1	Procarbazine HCl (Matulane)	257,76	DNA/RNA Synthesis
S2391	117-39-5	Quercetin (Sophoretin)	302,24	PI3K
S1526	950769-58-1	Quizartinib (AC220)	560,67	FLT3
S1533	841290-80-0	R406 (free base)	470,45	Syk
S2206	1025687-58-4	R935788 (Fostamatinib disodium, R788 disodium)	624,42	Syk
S1192	112887-68-0	Raltitrexed (Tomudex)	458,49	DNA/RNA Synthesis
S1799	95635-55-5	Ranolazine (Ranexa)	427,54	n.a.
S1039	53123-88-9	Rapamycin (Sirolimus)	914,18	Autophagy
S1178	755037-03-7	Regorafenib (BAY 73-4506)	482,82	c-Kit
S2821	48208-26-0	RG108	334,33	DNA Methyltransferase
S1362	1225497-78-8	Rigosertib (ON-01910)	473,47	PLK

S1153	186692-46-6	Roscovitine (Seliciclib, CYC202)	354,45	CDK
S2556	122320-73-4	Rosiglitazone (Avandia)	357,43	PPAR
S1098	459868-92-9	Rucaparib (AG-014699 , PF-01367338)	421,36	PARP
S1378	941678-49-5	Ruxolitinib (INCB018424)	306,37	JAK
S2352	53003-10-4	Salinomycin (Procoxacin)	751,00	Aromatase
S1006	379231-04-6	Saracatinib (AZD0530)	542,03	Bcr-Abl
S1076	152121-47-6	SB 203580	377,43	p38 MAPK
S1075	280744-09-4	SB 216763	371,22	GSK-3
S1067	301836-41-9	SB 431542	384,39	TGF-beta/Smad
S1476	356559-20-1	SB 525334	343,42	TGF-beta/Smad
S2182	940929-33-9	SB 743921	553,52	Kinesin
S2220	405554-55-4	SB590885	453,54	Raf
S1515	929016-96-6	SB939 (Pracinostat)	358,48	HDAC
S2198	1025065-69-3	SGI-1776 free base	405,42	Pim
S1112	1022150-57-7	SGX-523	359,41	c-Met
S1792	79902-63-9	Simvastatin (Zocor)	418,57	HMG-CoA Reductase
S2804	410536-97-9	Sirtinol	394,47	Sirtuin
S1145	345627-80-7	SNS-032 (BMS-387032)	380,53	CDK
S1154	1146618-41-8	SNS-314 Mesylate	527,04	Aurora Kinase
S1999	156-54-7	Sodium butyrate	110,09	HDAC
S1040	475207-59-1	Sorafenib (Nexavar)	637,03	PDGFR
S2791	425637-18-9	Sotrastaurin (AEB071)	438,48	PKC
S1129	1001645-58-4	SRT1720	506,02	Sirtuin
S2902	941685-37-6	S-Ruxolitinib	306,37	JAK
S1041	315702-99-9	STF-62247	267,35	Autophagy
S1312	18883-66-4	Streptozotocin (Zanosar)	265,22	DNA damage/DNA repair
S1080	658084-23-2	SU11274	568,09	c-Met
S1042	341031-54-7	Sunitinib Malate (Sutent)	532,56	c-Kit
S1108	761439-42-3	TAE684 (NVP-TAE684)	614,20	ALK
S2617	1035555-63-5	TAK-733	504,23	MEK
S2225	901-47-3	TAME	342,41	APC
S1972	54965-24-1	Tamoxifen Citrate (Nolvadex)	563,64	Autophagy
S1043	387867-13-2	Tandutinib (MLN518)	562,70	FLT3
S2231	332012-40-5	Telatinib (BAY 57-9352)	409,83	c-Kit
S1237	85622-93-1	Temozolomide	194,15	Autophagy
S1044	162635-04-3	Temsirolimus (Torisel)	1030,29	mTOR
S1787	29767-20-2	Teniposide (Vumon)	656,65	DNA damage/DNA repair
S2736	936091-26-8	TG101348 (SAR302503)	524,68	JAK
S1193	50-35-1	Thalidomide	258,23	E3 Ligase
S1577	948557-43-5	Tie2 kinase inhibitor	439,53	Tie-2
S1453	192185-72-1	Tipifarnib (Zarnestra)	489,40	Transferase
S1207	475108-18-0	Tivozanib (AV-951)	454,86	c-Kit
S2789	477600-75-2	Tofacitinib (CP-690550, Tasocitinib)	312,37	JAK
S1231	119413-54-6	Topotecan HCl	457,91	Topoisomerase

S1776	89778-27-8	Toremifene Citrate (Fareston, Acapodene)	598,08	Estrogen/progestogen Receptor
S2827	1222998-36-8	Torin 1	607,62	Autophagy
S2817	1223001-51-1	Torin 2	432,40	ATM/ATR
S1522	238750-77-1	Tosedostat (CHR2797)	406,47	Aminopeptidase
S2824	507475-17-4	TPCA-1	279,29	IκB/IKK
S1653	302-79-4	Tretinoin (Aberela)	300,40	Retinoid receptor
S1628	76-25-5	Triamcinolone Acetonide	434,50	Glucocorticoid receptor
S1045	58880-19-6	Trichostatin A (TSA)	302,40	HDAC
S1117	35943-35-2	Triciribine (Triciribine phosphate)	320,30	Akt
S3604	38748-32-2	Triptolide	360,40	NF-kB/MDM2
S1121	877877-35-5	TW-37	573,70	Bcl-2
S1591	58970-76-6	Ubenimex (Bestatin)	308,37	Aminopeptidase
S1168	1069-66-5	Valproic acid sodium salt (Sodium valproate)	166,19	Autophagy
S1046	443913-73-3	Vandetanib (Zactima)	475,35	VEGFR
S1101	212141-51-0	Vatalanib 2HCl (PTK787)	419,73	c-Kit
S1267	918504-65-1	Vemurafenib (PLX4032)	489,92	Raf
S1248	57-22-7	Vinblastine	824,96	AChR
S1241	2068-78-2	Vincristine	923,04	Autophagy
S2110	42971-09-5	Vinpocetine (Cavinton)	350,45	Sodium Channel
S1082	879085-55-9	Vismodegib (GDC-0449)	421,30	Hedgehog
S1047	149647-78-9	Vorinostat (SAHA)	264,30	Autophagy
S1048	639089-54-6	VX-680 (MK-0457, Tozasertib)	464,59	Aurora Kinase
S2694	629664-81-9	WAY-362450	438,47	FXR
S2243	856243-80-6	WP1130	384,27	Bcr-Abl
S1266	1062169-56-5	WYE-354	495,53	mTOR
S1173	1213269-23-8	WZ4002	494,18	EGFR
S1180	284028-89-3	XAV-939	312,31	Wnt/beta-catenin
S1118	956958-53-5	XL147	448,52	PI3K
S1523	1349796-36-6	XL765 (SAR245409)	599,66	mTOR
S1049	129830-38-2	Y-27632 2HCl	320,26	Autophagy
S1130	781661-94-7	YM155	443,29	E3 Ligase
S1219	371942-69-7	YM201636	467,48	PI3K
S2711	209984-56-5	YO-01027	463,48	Gamma-secretase
S1456	186497-07-4	Zibotentan (ZD4054)	424,43	Endothelin Receptor
S1443	111406-87-2	Zileuton	236,29	5-lipoxygenase
S1072	475110-96-4	ZSTK474	417,41	PI3K

Supplementary Table 2. List of antibodies used for Reverse-Phase Protein microArrays (RPPA) analysis.

Official Ab Name	Ab Name Reported on Dataset	Gene Name	Company	Catalog #	Species
14-3-3 beta	14-3-3-beta	YWHAB	Santa Cruz	sc-628	Rabbit
14-3-3 zeta	14-3-3-zeta	YWHAZ	Santa Cruz	sc-1019	Rabbit
4E-BP1	4E-BP1	EIF4EBP1	CST	9452	Rabbit
4E-BP1 (phospho S65)	4E-BP1_pS65	EIF4EBP1	CST	9456	Rabbit
53BP1	53BP1	TP53BP1	CST	4937	Rabbit
Acetyl CoA Carboxylase (phospho S79)	ACC_pS79	ACACA, ACACB	CST	3661	Rabbit
Acetyl CoA Carboxylase 1	ACC1	ACACA	Abcam	ab45174	Rabbit
ADAR1	ADAR1	ADAR	Abcam	ab88574	Mouse
Akt	Akt	AKT1,2,3	CST	4691	Rabbit
Akt (phospho S473)	Akt_pS473	AKT1,2,3	CST	9271	Rabbit
Akt (phospho T308)	Akt_pT308	AKT1,2,3	CST	2965	Rabbit
AMPK alpha	AMPKa	PRKAA1	CST	2532	Rabbit
AMPK alpha (phospho T172)	AMPKa_pT172	PRKAA1	CST	2535	Rabbit
AMPK alpha 2 (Phospho S345)	AMPK-a2_pS345	PRKAA2	Abcam	ab129081	Rabbit
Androgen Receptor	AR	AR	Abcam	ab52615	Rabbit
Annexin I	Annexin-I	ANXA1	BD Biosciences	610066	Mouse
Annexin VII	Annexin-VII	ANXA7	BD Biosciences	610668	Mouse
A-Raf	A-Raf	ARAF	CST	4432	Rabbit
ARID1A	ARID1A	ARID1A	Sigma-Aldrich	HPA005456	Rabbit
Atg3	Atg3	ATG3	CST	3415	Rabbit
Atg7	Atg7	ATG7	CST	8558	Rabbit
ATM	ATM	ATM	CST	2873	Rabbit
ATM (phospho S1981)	ATM_pS1981	ATM	CST	5883	Rabbit
ATR (Phospho S428)	ATR_pS428	ATR	Abcam	ab178407	Rabbit
Aurora B/AIM1	Aurora-B	AIM1	CST	3094	Rabbit
Axl	Axl	AXL	CST	8661	Rabbit
B7-H4	B7-H4	VTCN1	CST	14572	Rabbit
Bad (phospho S112)	Bad_pS112	BAD	CST	9291	Rabbit
Bak	Bak	BAK1	Abcam	ab32371	Rabbit
BAP1	BAP1	BAP1	Santa Cruz	sc-28383	Mouse
Bax	Bax	BAX	CST	2772	Rabbit
Bcl2	Bcl2	BCL2	Dako	M0887	Mouse
Bcl-xL	Bcl-xL	BCL2L1	CST	2762	Rabbit
Beclin	Beclin	BECN1	Santa Cruz	sc-10086	Goat
beta Actin	b-Actin	ACTB	CST	4970	Rabbit
beta Catenin	b-Catenin	CTNNB1	CST	9562	Rabbit

beta Catenin (phospho T41/S45)	b-Catenin_pT41_S45	CTNNB1	CST	9565	Rabbit
Bid	Bid	BID	CST	2002	Rabbit
Bim	Bim	BCL2L11	Abcam	ab32158	Rabbit
BiP/GRP78	BiP-GRP78	HSPA5	BD Biosciences	610978	Mouse
B-Raf	B-Raf	BRAF	CST	14814	Rabbit
B-Raf (phospho S445)	B-Raf_pS445	BRAF	CST	2696	Rabbit
BRD4	BRD4	BRD4	CST	13440	Rabbit
c-Abl	c-Abl	ABL	CST	2862	Rabbit
c-IAP2	c-IAP2	BIRC3	CST	3130	Rabbit
Caspase-3 active	Caspase-3	CASP3	Abcam	ab32042	Rabbit
Caspase-7 (cleaved D198)	Caspase-7-cleaved	CASP7	CST	9491	Rabbit
Caspase-8	Caspase-8	CASP8	CST	9746	Mouse
Caveolin-1	Caveolin-1	CAV1	CST	3238	Rabbit
CD4	CD4	CD4	Abcam	ab133616	Rabbit
CD20	CD20	MS4A1	Abcam	ab78237	Rabbit
CD29	CD29	ITGB1	BD Biosciences	610467	Mouse
CD31	CD31	PECAM1	Dako	M0823	Mouse
CD44	CD44	CD44	CST	3570	Mouse
CD45	CD45	CD45	DAKO	M070129-2	Mouse
CD49b	CD49b	ITGA2	BD Biosciences	611016	Mouse
CD134/OX40	CD134	CD134	Abcam	ab76000	Rabbit
cdc2 (Phospho Y15)	cdc2_pY15	CDK1	CST	4539	Rabbit
cdc25C	cdc25C	CDC25C	CST	4688	Rabbit
CDK1	CDK1	CDK1	Abcam	ab32384	Rabbit
CDKN2A/p16INK4a	p16INK4a	CDKN2A	Abcam	ab81278	Rabbit
Chk1	Chk1	CHEK1	CST	2360	Mouse
Chk1 (phospho S296)	Chk1_pS296	CHEK1	Abcam	ab79758	Rabbit
Chk2	Chk2	CHEK2	CST	3440	Mouse
Chk2 (phospho T68)	Chk2_pT68	CHEK2	CST	2197	Rabbit
c-Jun (phospho S73)	c-Jun_pS73	JUN	CST	9164	Rabbit
c-Kit	c-Kit	KIT	Abcam	ab32363	Rabbit
Claudin 7	Claudin-7	CLDN7	Novus Biologicals	NB100-91714	Rabbit
c-Met (phospho Y1234/Y1235)	c-Met_pY1234_Y1235	MET	CST	3129	Rabbit
c-Myc	c-Myc	MYC	Santa Cruz	sc-764	Rabbit
COG3	COG3	COG3	ProteinTech	11130-1-AP	Rabbit
COL6A1	Collagen-VI	COL6A1	Santa Cruz	sc-20649	Rabbit
Connexin 43	Connexin-43	CNST43	CST	3512	Rabbit
Cox2	Cox2	PTGS2	CST	4842	Rabbit
Cox-IV	Cox-IV	PTGS3	CST	4850	Rabbit
C-Raf (phospho S338)	C-Raf_pS338	RAF1	CST	9427	Rabbit
C-Raf/Raf-1	C-Raf	RAF1	Millipore	04-739	Rabbit
CREB	CREB	CREB1	CST	9197	Rabbit
Cyclin B1	Cyclin-B1	CCNB1	Epitomics	1495-1	Rabbit
Cyclin D1	Cyclin-D1	CCND1	Santa Cruz	sc-718	Rabbit

Cyclin D3	Cyclin-D3	CCND3	CST	2936	Mouse
Cyclin E1	Cyclin-E1	CCNE1	Santa Cruz	sc-247	Mouse
Cyclophilin F	Cyclophilin-F	PPIF	Abcam	ab110324	Mouse
Detyrosinated alpha-Tubulin	D-a-Tubulin	TUBA1A	Abcam	ab48389	Rabbit
Dimethyl-Histone H3 (Lys4)	DM-Histone-H3	HISTH3	Millipore	07-030	Rabbit
Dimethyl-K9 Histone H3	DM-K9-Histone-H3	H3K9ME2	Abcam	ab32521	Rabbit
DUSP4/MKP2	DUSP4	DUSP4	CST	5149	Rabbit
E2F-1	E2F1	E2F1	Santa Cruz	sc-251	Mouse
E-Cadherin	E-Cadherin	CDH1	CST	3195	Rabbit
eEF2	eEF2	EEF2	CST	2332	Rabbit
eEF2K	eEF2K	EEF2K	CST	3692	Rabbit
EGFR	EGFR	EGFR	CST	2232	Rabbit
EGFR (phospho Y1173)	EGFR_pY1173	EGFR	Abcam	ab32578	Rabbit
eIF4E	eIF4E	EIF4E	CST	9742	Rabbit
eIF4E (Phospho S209)	eIF4E_pS209	EIF4E	Abcam	ab76256	Rabbit
eIF4G	eIF4G	EIF4G1	CST	2498	Rabbit
Elk1 (phospho S383)	Elk1_pS383	ELK1	CST	9181	Rabbit
ENY2	ENY2	ENY2	GeneTex	GTX629542	Mouse
Epithelial Membrane Antigen	EMA	EMA	Dako	M061329-2	Mouse
ErbB2/HER2	HER2	ERBB2	Lab Vision	MS-325-P1	Mouse
ErbB2/HER2 (phospho Y1248)	HER2_pY1248	ERBB2	R&D Systems	AF1768	Rabbit
ErbB3/HER3	HER3	ERBB3	Santa Cruz	sc-285	Rabbit
ErbB3/HER3 (phospho Y1289)	HER3_pY1289	ERBB3	CST	4791	Rabbit
ERCC1	ERCC1	ERCC1	Santa Cruz	sc-17809	Mouse
ERCC5	ERCC5	ERCC5	ProteinTech	11331-1-AP	Rabbit
ERRFI1/MIG6	MIG6	ERRFI1	Sigma-Aldrich	WH0054206M1	Mouse
Estrogen Receptor	ER	ESR1	Lab Vision	RM-9101	Rabbit
Estrogen Receptor alpha (Phospho S118)	ER-a_pS118	ESR1	Abcam	ab32396	Rabbit
Ets-1	Ets-1	ETS1	Bethyl	A303-501A	Rabbit
FAK	FAK	PTK2	Abcam	ab40794	Rabbit
FAK (phospho Y397)	FAK_pY397	PTK2	CST	3283	Rabbit
Fatty Acid Synthase	FASN	FASN	CST	3180	Rabbit
Fibronectin	Fibronectin	FN1	Epitomics	1574-1	Rabbit
FoxM1	FoxM1	FOXM1	CST	5436	Rabbit
FoxO3a	FoxO3a	FOXO3	CST	2497	Rabbit
FoxO3a (phospho S318/S321)	FoxO3a_pS318_S321	FOXO3	CST	9465	Rabbit
G6PD	G6PD	G6PD	CST	8866	Rabbit
Gab2	Gab2	GAB2	CST	3239	Rabbit
GAPDH	GAPDH	GAPDH	Life Technologies	AM4300	Mouse
GATA3	GATA3	GATA3	BD Biosciences	558686	Mouse
GCLM	GCLM	GCLM	Abcam	ab124827	Rabbit
GCN5L2	GCN5L2	KAT2A	CST	3305	Rabbit

Glutamate Dehydrogenase1/2	Glutamate-D1-2	GLUD	CST	12793	Rabbit
Glutaminase	Glutaminase	GLS	Abcam	ab156876	Rabbit
Glycogen Synthase	Gys	GYS1	CST	3886	Rabbit
Glycogen Synthase (phospho S641)	Gys_pS641	GYS1	CST	3891	Rabbit
Granzyme B	Granzyme-B	GZMB	CST	4275	Rabbit
GSK-3alpha/beta	GSK-3a-b	GSK3A, GSK3B	Santa Cruz	sc-7291	Mouse
GSK-3alpha/beta (phospho S21/S9)	GSK-3a-b_pS21_S9	GSK3A, GSK3B	CST	9331	Rabbit
H2AX (phospho S140)	H2AX_pS140	H2AX	Pierce Biotechnology	MA1-2022	Mouse
Heregulin	Heregulin	NRG1	CST	2573	Rabbit
HES1	HES1	HES1	CST	11988	Rabbit
Hexokinase II	Hexokinase-II	HK2	CST	2867	Rabbit
Hif-1 alpha	Hif-1-alpha	HIF1A	BD Biosciences	610958	Mouse
Histone H3	Histone-H3	H3F3A, H3F3B	Abcam	ab1791	Rabbit
HLA-DR/DP/DQ/DX	HLA-DR-DP-DQ-DX	HLA	Santa Cruz	sc-53302	Mouse
HSP27	HSP27	HSP27	CST	2402	Mouse
HSP27 (phospho S82)	HSP27_pS82	HSBP1	CST	2401	Rabbit
HSP70	HSP70	HSP70	CST	4872	Rabbit
IGF1R (phospho Y1135/Y1136)	IGF1R_pY1135_Y1136	IGF1R	CST	3024	Rabbit
IGFBP2	IGFBP2	IGFBP2	CST	3922	Rabbit
IGFRb	IGFRb	INSR	CST	3027	Rabbit
INPP4b	INPP4b	INPP4B	CST	4039	Rabbit
Insulin Receptor beta	IR-b	INSRB	CST	3025	Rabbit
IRF-1	IRF-1	IRF1	Santa Cruz	sc-497	Rabbit
IRS1	IRS1	IRS1	Millipore	06-248	Rabbit
Jagged1	Jagged1	JAG1	Abcam	ab109536	Rabbit
Jak2	Jak2	JAK2	CST	3230	Rabbit
JNK/SAPK (phospho T183/Y185)	JNK_pT183_Y185	MAPK8	CST	4668	Rabbit
JNK2	JNK2	MAPK9	CST	4672	Rabbit
LC3A/B	LC3A-B	LC3AB	CST	4108	Rabbit
Lck	Lck	LCK	CST	2752	Rabbit
LDHA	LDHA	LDHA	CST	3582	Rabbit
LRP6 (phospho S1490)	LRP6_pS1490	LRP6	CST	2568	Rabbit
MAPK (phospho T202/Y204)	MAPK_pT202_Y204	MAPK1, MAPK3	CST	4377	Rabbit
Mcl 1	Mcl-1	MCL1	CST	5453	Rabbit
MDM2 (phospho S166)	MDM2_pS166	MDM2	CST	3521	Rabbit
MEK1	MEK1	MAP2K1	Abcam	ab32576	Rabbit
MEK1 (phospho S217/S221)	MEK1_pS217_S221	MAP2K1 MAP2K2	CST	9154	Rabbit
MERIT40 (Phospho S29)	MERIT40_pS29	BABAM1	CST	12110	Rabbit
Merlin/NF2	Merlin	NF2	Novus Biologicals	22710002	Rabbit
MIF	MIF	MIF	Santa Cruz	sc-20121	Rabbit

MMP2	MMP2	MMP2	CST	4022	Rabbit
Mnk1	Mnk1	MKNK1	CST	2195	Rabbit
Monocarboxylic Acid Transporter 4	MCT4	SLC16A4	Millipore	AB3314P	Rabbit
MSH6	MSH6	MSH6	Novus Biologicals	22030002	Rabbit
MSI2	MSI2	MSI2	Abcam	ab76148	Rabbit
mTOR	mTOR	MTOR	CST	2983	Rabbit
mTOR (phospho S2448)	mTOR_pS2448	MTOR	CST	2971	Rabbit
Myosin heavy chain 11	Myosin-11	MYH11	Novus Biologicals	21370002	Rabbit
Myosin IIa (phospho S1943)	Myosin-IIa_pS1943	MYH9	CST	5026	Rabbit
Myt1	Myt1	MYT1	CST	4282	Rabbit
NAPSIN A	NAPSIN-A	NAPSA	Abcam	ab129189	Rabbit
N-Cadherin	N-Cadherin	CDH2	CST	4061	Rabbit
NDRG1 (phospho T346)	NDRG1_pT346	NDRG1	CST	3217	Rabbit
NDUFB4	NDUFB4	NDUFB4	Abcam	ab110243	Mouse
NF-kappaB p65 (phospho S536)	NF-kB-p65_pS536	RELA	CST	3033	Rabbit
Notch1	Notch1	NOTCH1	CST	3268	Rabbit
Notch3	Notch3	NOTCH3	Santa Cruz	sc-5593	Rabbit
N-Ras	N-Ras	NRAS	Santa Cruz	sc-31	Mouse
Oct-4	Oct-4	OCT4	CST	2750	Rabbit
p21	p21	CDKN1A	Santa Cruz	sc-397	Rabbit
p27 KIP 1	p27-Kip-1	CDKN1B	Abcam	ab32034	Rabbit
p27/KIP 1 (phospho T198)	p27_pT198	CDKN1B	Abcam	ab64949	Rabbit
p38 MAPK	p38	MAPK14	CST	9212	Rabbit
p38 MAPK (phospho T180/Y182)	p38_pT180_Y182	MAPK14	CST	9211	Rabbit
p44/42 MAPK	p44-42-MAPK	MAPK3	CST	4695	Rabbit
p53	p53	TP53	CST	9282	Rabbit
p70 S6 Kinase (phospho T389)	p70-S6K_pT389	RPS6KB1	CST	9205	Rabbit
p70/S6K1	p70-S6K1	RPS6KB1	Abcam	ab32529	Rabbit
p90RSK (phospho T573)	p90RSK_pT573	RPS6K	CST	9346	Rabbit
PAI-1	PAI-1	SERPINE1	BD Biosciences	612024	Mouse
PAICS	PAICS	PAICS	Sigma-Aldrich	HPA035895	Rabbit
PAK1	PAK1	PAK1	CST	2602	Rabbit
PAK4	PAK4	PAK4	CST	3242	Rabbit
PAR	PAR	PAR	Trevigen	4336-BPC-100	Rabbit
PARK7/DJ1	DJ1	PARK7	Abcam	ab76008	Rabbit
PARP	PARP	PARP1	CST	9532	Rabbit
Paxillin	Paxillin	PXN	Epitomics	1500-1	Rabbit
P-Cadherin	P-Cadherin	CDH3	CST	2130	Rabbit
PCNA	PCNA	PCNA	CST	2586	Mouse
PD-1	PD-1	PD1	CST	43248	Rabbit
Pdcd4	Pdcd4	PDCD4	Rockland	600-401-965	Rabbit
PDGFR beta	PDGFR-b	PDGFRB	CST	3169	Rabbit

PDHK1	PDHK1	PDHK1	CST	3820	Rabbit
PDK1	PDK1	PDPK1	CST	3062	Rabbit
PDK1 (phospho S241)	PDK1_pS241	PDPK1	CST	3061	Rabbit
PD-L1	PD-L1	CD274	CST	13684	Rabbit
PEA-15	PEA-15	PEA15	CST	2780	Rabbit
PED/PEA-15 (phospho S116)	PEA-15_pS116	PEA15	Invitrogen	44-836G	Rabbit
PI3 Kinase p110 alpha	PI3K-p110-a	PIK3CA	CST	4255	Rabbit
PI3K p110 beta	PI3K-p110-b	PIK3CB	Santa Cruz	sc-376412	Mouse
PI3K p85	PI3K-p85	PIK3R1	Millipore	06-195	Rabbit
PKA RI alpha	PKA-a	PRKAR1A	CST	5675	Rabbit
PKCalpha	PKCa	PRKCA	CST	2056	Rabbit
PKC beta II (phospho S660)	PKC-b-II_pS660	PRKCA, PRKCB PRKCD, PRKCE PRKCH, PRKCQ	CST	9371	Rabbit
PKC delta (phospho S664)	PKC-delta_pS664	PRKCD	Millipore	07-875	Rabbit
PKM2	PKM2	PKM2	CST	4053	Rabbit
PLC gamma2 (phospho Y759)	PLC-gamma2_pY759	PLCG2	CST	3874	Rabbit
PLK1	PLK1	PLK1	CST	4513	Rabbit
PMS2	PMS2	PMS2	Novus Biologicals	22510002	Rabbit
PRAS40	PRAS40	AKT1S1	Invitrogen	AHO1031	Mouse
PRAS40 (phospho T246)	PRAS40_pT246	AKT1S1	Life Technologies	441100G	Rabbit
PREX1	PREX1	PREX1	Abcam	ab102739	Rabbit
Progesterone Repector	PR	PGR	Abcam	ab32085	Rabbit
PTEN	PTEN	PTEN	CST	9552	Rabbit
Rab11	Rab11	RAB11A,B	CST	3539	Rabbit
Rab25	Rab25	RAB25	CST	4314	Rabbit
Rad50	Rad50	RAD50	Millipore	05-525	Mouse
Rad51	Rad51	RAD51	CST	8875	Rabbit
Raptor	Raptor	RPTOR	CST	2280	Rabbit
Rb	Rb	RB1	CST	9309	Mouse
Rb (phospho S807/S811)	Rb_pS807_S811	RB1	CST	9308	Rabbit
RBM15	RBM15	RBM15	Novus Biologicals	21390002	Rabbit
Rheb	Rheb	RHEB	R&D Systems	MAB3426	Mouse
Rictor	Rictor	RICTOR	CST	2114	Rabbit
Rictor (phospho T1135)	Rictor_pT1135	RICTOR	CST	3806	Rabbit
RIP	RIP	RIP	CST	4926	Rabbit
RPA32	RPA32	RPA32	CST	2208	Rat
RPA32 (Phospho S4/S8)	RPA32_pS4_S8	RPA32	Bethyl	A300-245A	Rabbit
RSK	RSK	RPS6KA1 RPS6KA2 RPS6KA3	CST	9347	Rabbit
S6 (phospho S235/S236)	S6_pS235_S236	RPS6	CST	2211	Rabbit
S6 (phospho S240/S244)	S6_pS240_S244	RPS6	CST	2215	Rabbit

S6 Ribosomal Protein	S6	RPS6	CST	2317	Mouse
SCD	SCD	SCD	Santa Cruz	sc-58420	Mouse
SDHA	SDHA	SDHA	CST	11998	Rabbit
SF2/ASF	SF2	SRSF1	Invitrogen	32-4500	Mouse
Shc (phospho Y317)	Shc_pY317	SHC1	CST	2431	Rabbit
SHP-2 (phospho Y542)	SHP-2_pY542	PTPN11	CST	3751	Rabbit
SLC1A5	SLC1A5	SLC1A5	Sigma-Aldrich	HPA035240	Rabbit
Slfn11	Slfn11	SLFN11	Santa Cruz	sc-136891	Goat
Smac/Diablo	Smac	DIABLO	CST	2954	Mouse
Smad1	Smad1	SMAD1	Abcam	ab33902	Rabbit
Smad3	Smad3	SMAD3	Abcam	ab40854	Rabbit
Smad4	Smad4	SMAD4	Santa Cruz	sc-7966	Mouse
Snail	Snail	SNAI1	CST	3895	Mouse
SOD1	SOD1	SOD1	CST	4266	Mouse
SOD2	SOD2	SOD2	CST	13141	Rabbit
Sox2	Sox2	SOX2	CST	2748	Rabbit
Src	Src	SRC	Millipore	05-184	Mouse
Src (phospho Y527)	Src_pY527	SRC, YES1, FYN FGR	CST	2105	Rabbit
Src Family (phospho Y416)	Src_pY416	SRC, LYN, FYN LCK, YES1, HCK	CST	2101	Rabbit
Stat3	Stat3	STAT3	CST	4904	Rabbit
Stat3 (phospho Y705)	Stat3_pY705	STAT3	CST	9145	Rabbit
Stat5a	Stat5a	STAT5A	Abcam	ab32043	Rabbit
Stathmin 1	Stathmin-1	STMN1	Abcam	ab52630	Rabbit
Syk	Syk	SYK	Santa Cruz	sc-1240	Mouse
Tau	Tau	TAU	Millipore	05-348	Mouse
TAZ	TAZ	WWTR1	CST	4883	Rabbit
TFAM	TFAM	TFAM	CST	7495	Rabbit
TIGAR	TIGAR	C12ORF5	Abcam	ab137573	Rabbit
Transferrin Receptor	TFRC	TFRC	Novus Biologicals	22500002	Rabbit
Transglutaminase II	Transglutaminase	TGM2	Lab Vision	MS-224-P1	Mouse
TRIM25	TRIM25	TRIM25	Abcam	ab167154	Rabbit
TSC1/Hamartin	TSC1	TSC1	CST	4906	Rabbit
TSC2/Tuberin (phospho T1462)	Tuberin_pT1462	TSC2	CST	3617	Rabbit
TTF1**	TTF1	NKX2-1	Abcam	ab76013	Rabbit
Tuberin	Tuberin	TSC2	Abcam	ab32554	Rabbit
TUFM	TUFM	TUFM	Abcam	ab173300	Rabbit
Twist	TWIST	TWIST2	Santa Cruz	sc-81417	Mouse
Tyro3	Tyro3	TYRO3	CST	5585	Rabbit
UBAC1	UBAC1	UBAC1	Sigma-Aldrich	HPA005651	Rabbit
Ubiquityl Histone H2B	Ubq-Histone-H2B	HIST1H2BB	CST	5546	Rabbit
UGT1A	UGT1A	UGT1A1	Santa Cruz	sc-271268	Mouse
ULK1 (phospho S757)	ULK1_pS757	ULK1	CST	6888	Rabbit
VASP	VASP	VASP	CST	3112	Rabbit

VDAC1/Porin	Porin	VDAC1	Abcam	ab14734	Mouse
VEGF Receptor 2	VEGFR-2	KDR	CST	2479	Rabbit
VHL/EPPK1**	VHL-EPPK1	EPPK1	BD Biosciences	556347	Mouse
Vimentin	Vimentin	VIM	Dako	M0725	Mouse
Wee1	Wee1	WEE1	CST	4936	Rabbit
Wee1 (Phospho S642)	Wee1_pS642	WEE1	CST	4910	Rabbit
WIPI1	WIPI1	WIPI1	CST	12124	Rabbit
WIPI2	WIPI2	WIPI2	CST	8567	Rabbit
XBP1	XBP1	XBP1	Santa Cruz	sc-32136	Goat
XPA	XPA	XPA	Santa Cruz	sc-56813	Mouse
XPF	XPF	XPF	Abcam	ab73720	Rabbit
XRCC1	XRCC1	XRCC1	CST	2735	Rabbit
YAP	YAP	YAP1	Santa Cruz	sc-15407	Rabbit
YAP (phospho S127)	YAP_pS127	YAP1	CST	4911	Rabbit
YB1 (phospho S102)	YB1_pS102	YBX1	CST	2900	Rabbit
ZAP-70	ZAP-70	ZAP70	CST	2705	Rabbit

Supplementary Table 3. Patient and GSC line characteristics.

Case Code	Patients													Cell lines		
	Age/ Sex	KPS	Sympt. (mo.)	Prim Rec	Location	Ki 67 (%)	MGMT	EGFRvIII	PTEN	VEGF	PFS (mo.)	OS (mo.)	CD133 (%)	SOX2 (%)	Estimated Stem cell frequency
1	40/M	80	2.5	P	Temporal	20	M	negative	normal	iper	6.0	12.5	95.8	95.2	4.3
61	59/M	80	2.0	P	Occipital	35	UM	positive	normal	normal	3.0	6.0	2.5	81.6	1.6
83	52/M	70	0.5	P	Temporal	40	UM	positive	normal	iper	3.0	8.0	0.2	77.7	2.66
163	56/M	50	5.0	P	Parietal	12	UM	negative	ipo	normal	1.0	2.0	1.2	89.2	2.33

KPS, Karnofsky Performance Status; Sympt., symptom duration; Prim., primary tumor; Rec., recurrent tumor; PFS, progression-free survival; OS, overall survival.

Supplementary Table 4. List of genes corresponding to significant antibodies and grouped using the Venn diagram in Figure 4C.

n	GSCs	GdECs	Common
1	KDR	FASN	RPS6
2	CAV1	H3K9ME2	CASP3
3	BIRC3	PTK2	ITGB1
4	NF2	KIT	XPA
5	ERBB3	XRCC1	PECAM1 6
7	CTNNB1	SRC	
8	PRKAR1A	YES1	
9	CDK1	FYN	
10	CD274	FGR	
11	ENY2	PDGFRB	
12	ACTB	TP53BP1	
13	EGFR	COL6A1	
14	PAR	PLCG2	
15	TGM2	RELA	
16	FOXO3	CREB1	
17	ATR	CHEK1	
18	MAPK8	E2F1	
19	HIST1H2BB	LYN	
20	VTCN1	LCK	
21	IGF1R	HCK	
22	BAX	TSC1	
23		BCL2L11	
24		NOTCH3	
25		OCT4	
26		BECN1	
27		EIF4EBP1	
28		NDUFB4	
29		PIK3CB	
30		PDPK1	
31		H3F3A	
32		H3F3B	
33		CD4	
34		RPA32	
35		CASP7	

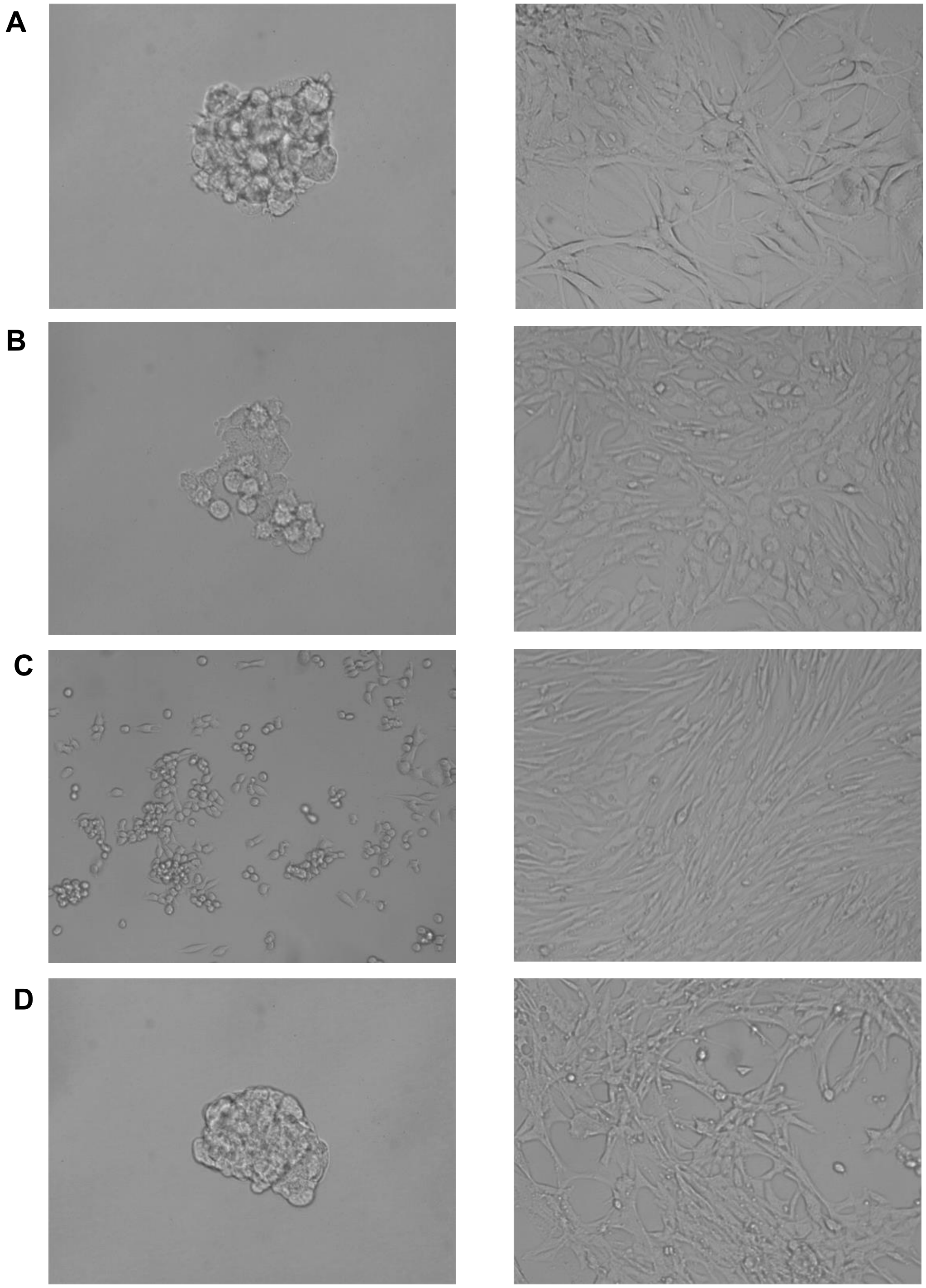

Supplementary Figure S1. A-D. Morphological changes of the four GSC lines used in the study (**A**, GSC#1; **B**, GSC#61; **C,** GSC#83; **D**, GSC#163) after being induced to transdifferentiate for 2 weeks. *Left panel*, tumorspheres in stem cell medium; *right panel*, net-like structures under endothelial conditions (magnification 10X).

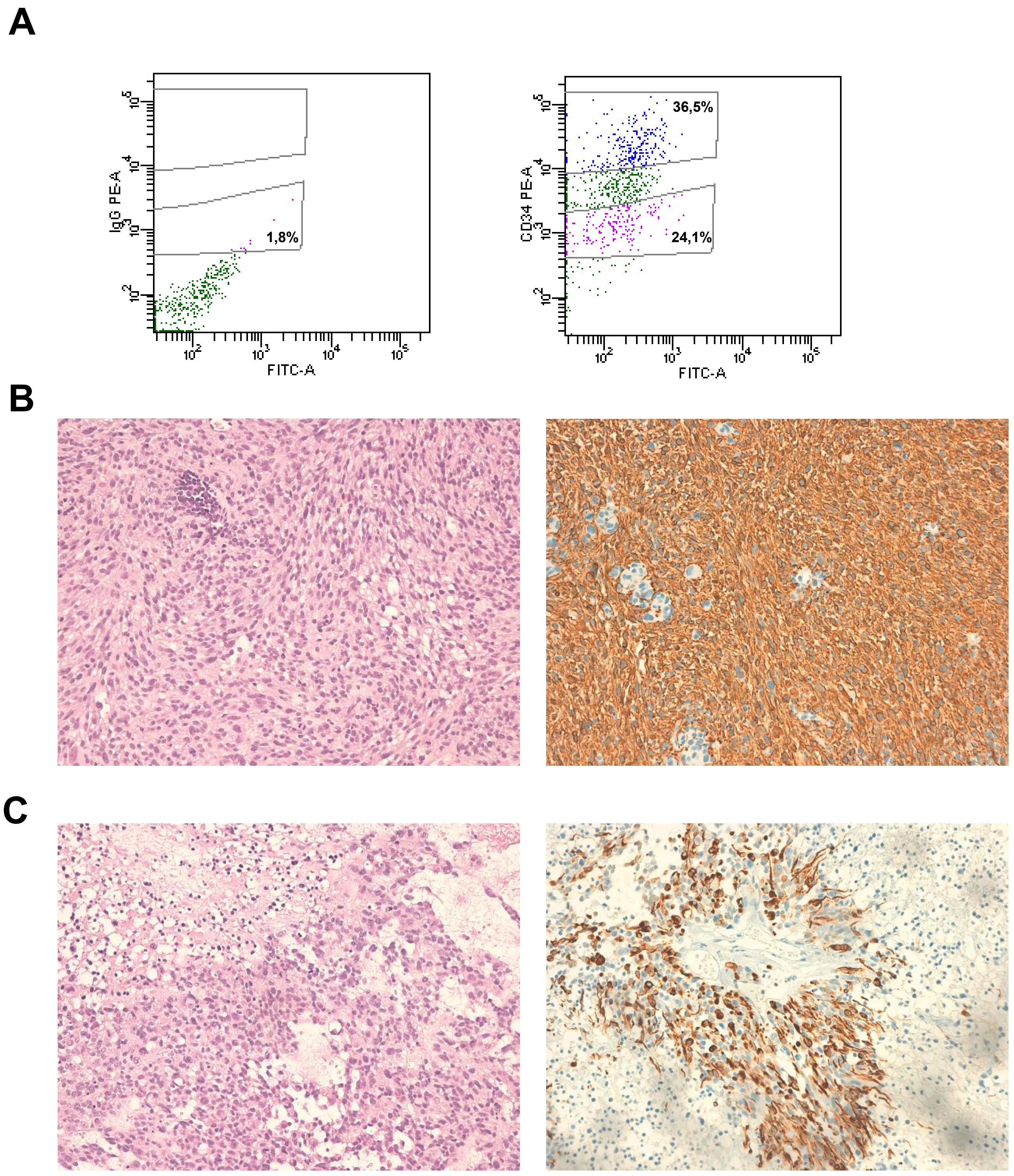

Supplementary Figure S2. (**A**) Fluorescent-activated cell sorting dot plots of CD34$^{-/low}$ and CD34^{high} GSC#163 after two weeks of culture in endothelial conditions under hypoxia. Percentage and squares indicate the sorted subpopulations of cells with different CD34-expression levels (*left*, IgG$_1$ isotype control sample; *right,* CD34 sample). (**B-C**) Immunohistochemical analysis of CD34^{low} (B) and CD34^{high} (C) GdEC subcutaneous tumor xenografts based on the expression of the astrocytic marker glial fibrillary acidic protein (GFAP, *right panels*), showing tumors with different levels of differentiation. (*Left panels*, haematoxylin and eosin staining; magnification 200X).

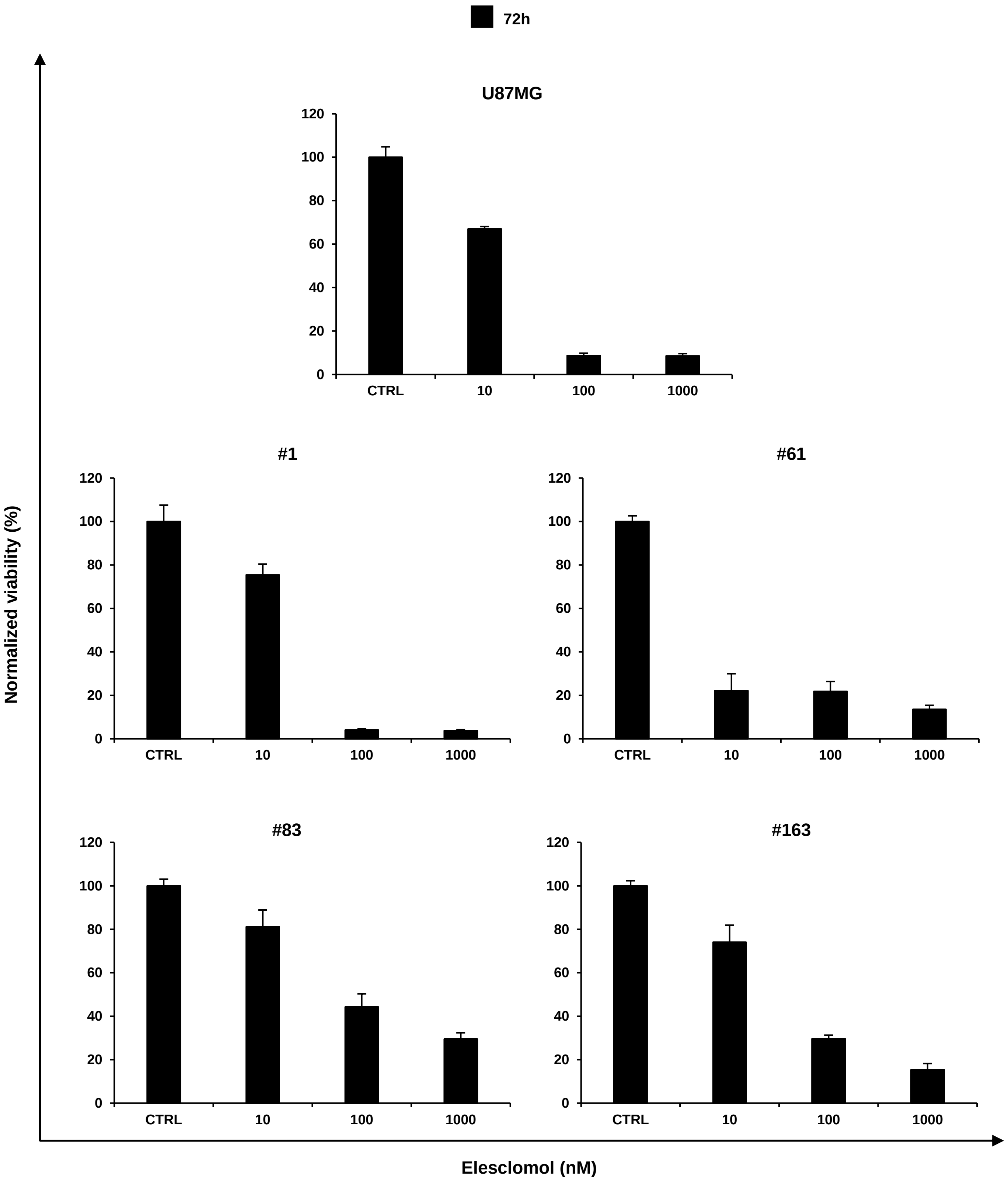

Supplementary Figure S3. Concentration-response assays on U87MG and all the four glial cell lines derived from the selected GSC lines.

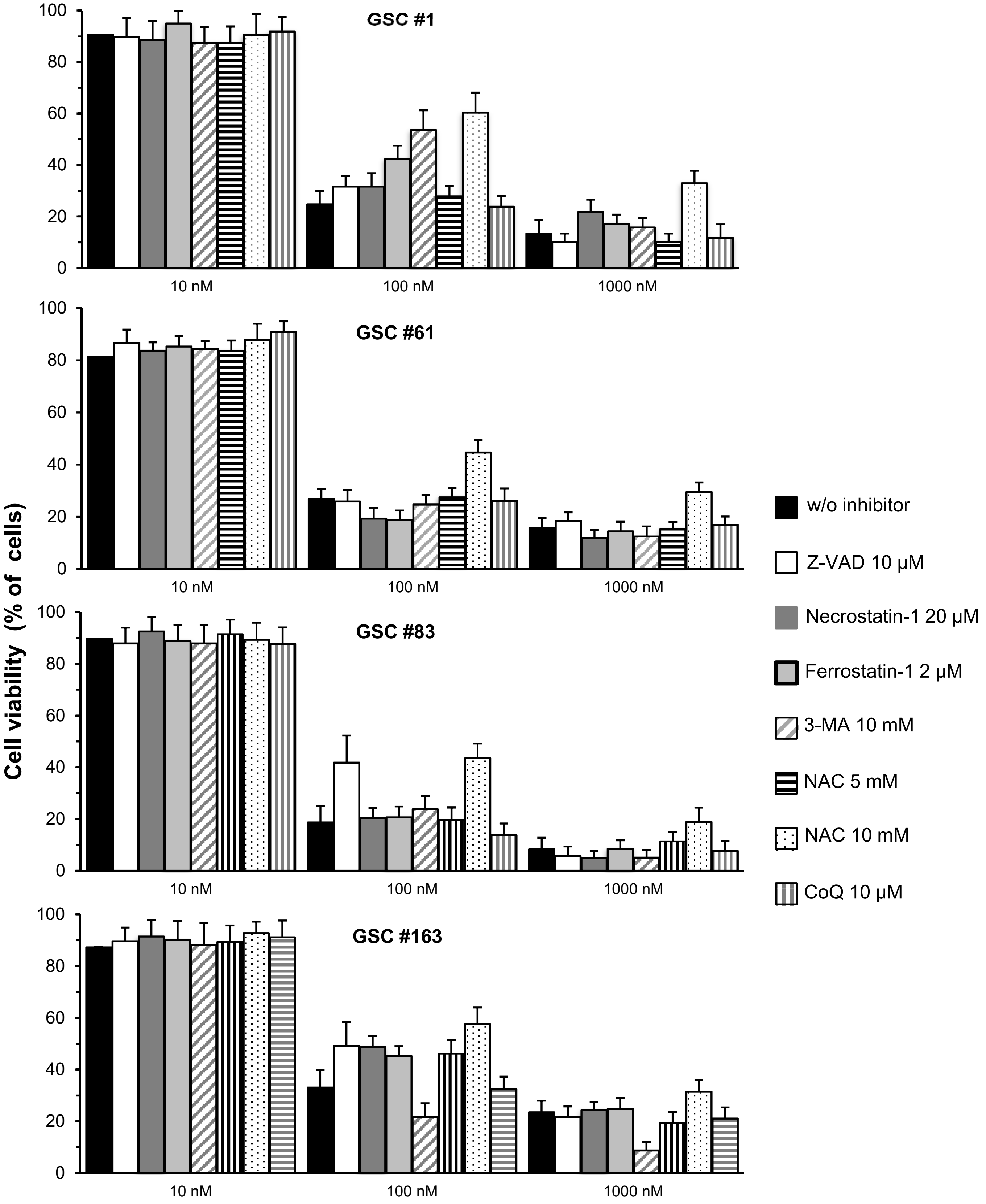

Supplementary Figure S4. Cytofluorimetric cell-by-cell analysis of viability in four different GSC lines treated with 10, 100, or 1000 nM elesclomol in the presence or absence of the following cell death inhibitors: z-VAD, necrostatin-1, ferrostatin-1, 3-MA, NAC, and CoQ at the indicated concentrations. Results obtained from four independent experiments are expressed as percentage *vs* control untreated cells and reported as means ± SD.

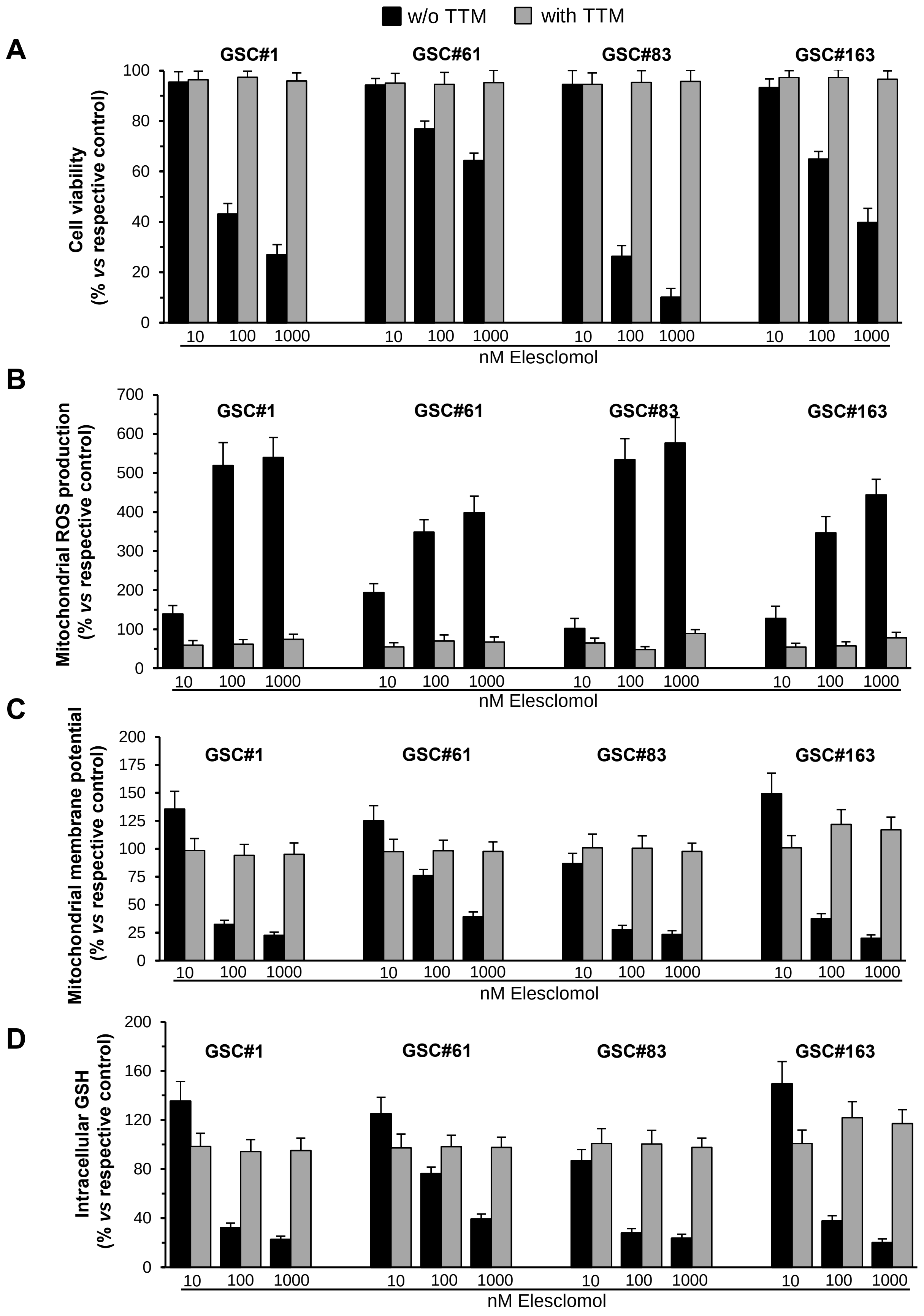

Supplementary Figure S5. Cytofluorimetric cell-by-cell analysis of cell viability (**A**), mitochondrial ROS production (**B**), mitochondrial membrane potential (**C**), and GSH (**D**) in four different GSC lines treated with 10, 100, or 1000 nM elesclomol in the presence or absence of the copper chelating agent TTM. Results obtained from four independent experiments are expressed as percentage vs control untreated cells and reported as means ± SD.

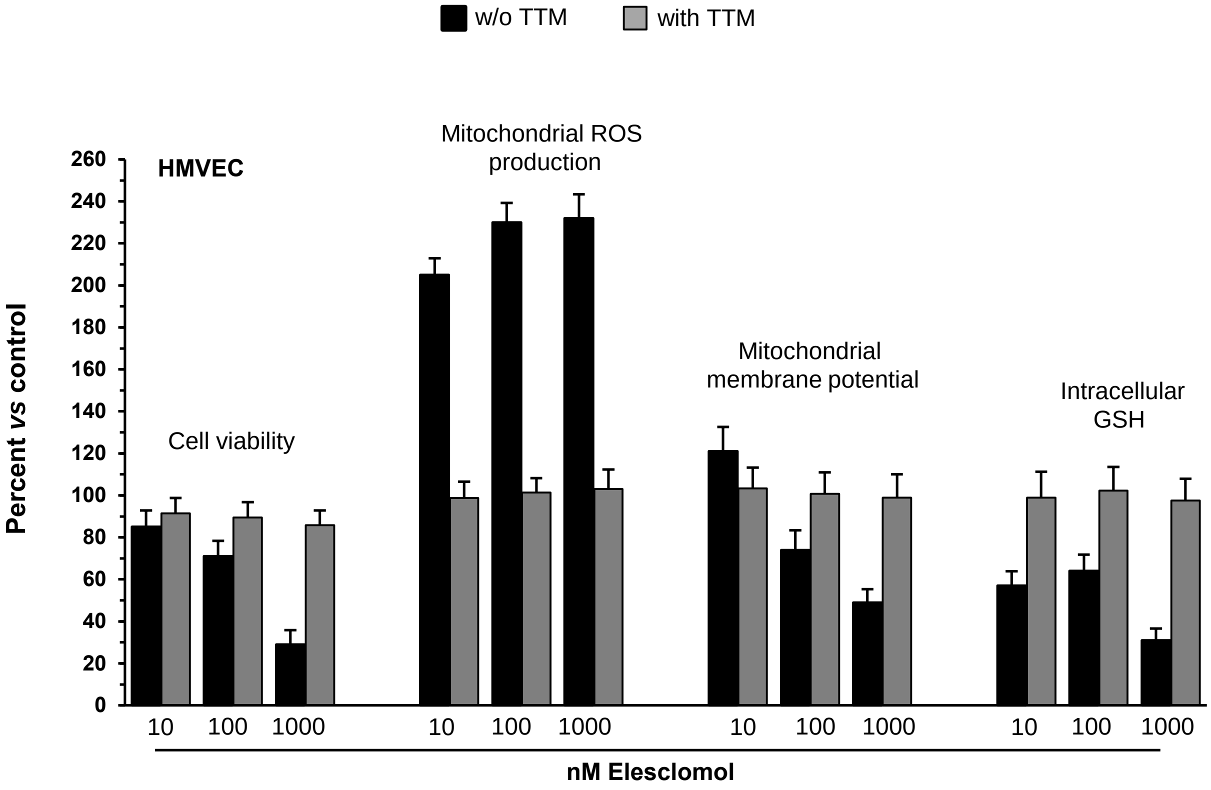

Supplementary Figure S6. Cytofluorimetric cell-by-cell analysis of cell viability, mitochondrial ROS production, mitochondrial membrane potential, and GSH in HMVECs, used as a control of non-tumoral endothelial cell line, treated with 10, 100, or 1000 nM Elesclomol in the presence or absence of the copper chelating agent TTM. Results obtained from four independent experiments are expressed as percentage *vs* control untreated cells and reported as means ± SD.

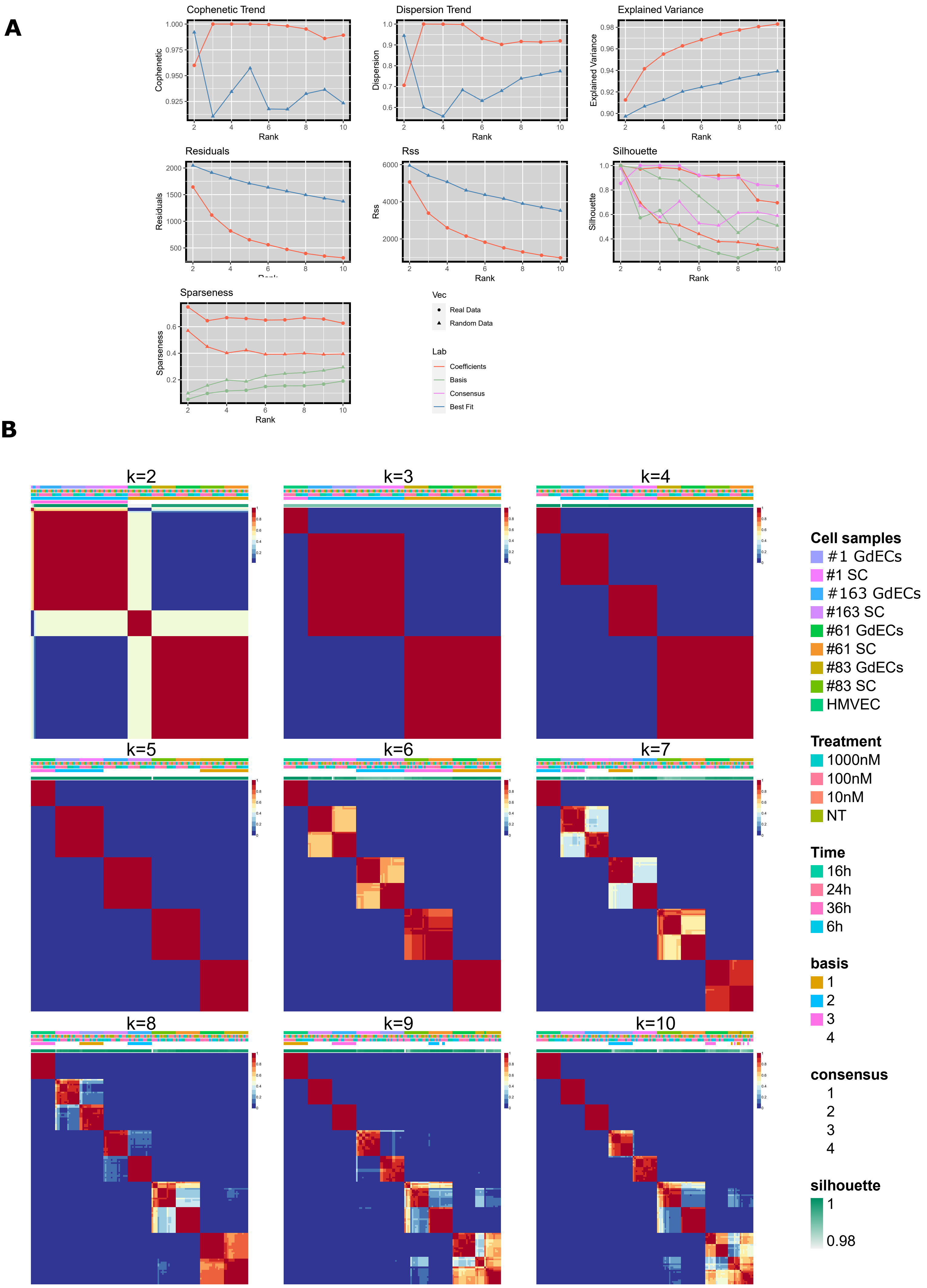

Supplementary Figure S7. Illustration of the rationale suitable for the choice of rank k, a critical parameter that defines the number of metagenes used to approximate the target matrix (Gaujoux & Seoighe, 2010). **A**) Measurements are applied to both real data (**circles**) and randomized data (**triangles**). The rationale for choosing rank stems on diverse metrics, **i**) trend of the cophenetic coefficient: Brunet et al. (2004) suggest choosing the smallest value of k for which there is a decrease in the trend of the cophenetic; **ii**) trend of the dispersion coefficient introduced by Kim & Park. (2007); **iii**) explained variance by increasing rank; **iv**) trend of residuals; **v**) trend of RSS: Hutchins et al. (2008) suggest taking the first rank value for which we have an inflection point. Frigyesi et al. (2008) instead consider the first rank value for which the decrease of the RSS on real data is less than the decrease of the RSS on the random data; **vi**) silhouette values measured on the matrices of the base, of the coefficients and the consensus matrix; **vii**) trend of the sparseness introduced by Hoyer (2004). **B**) Multiple consensus maps corresponding to different value of k.

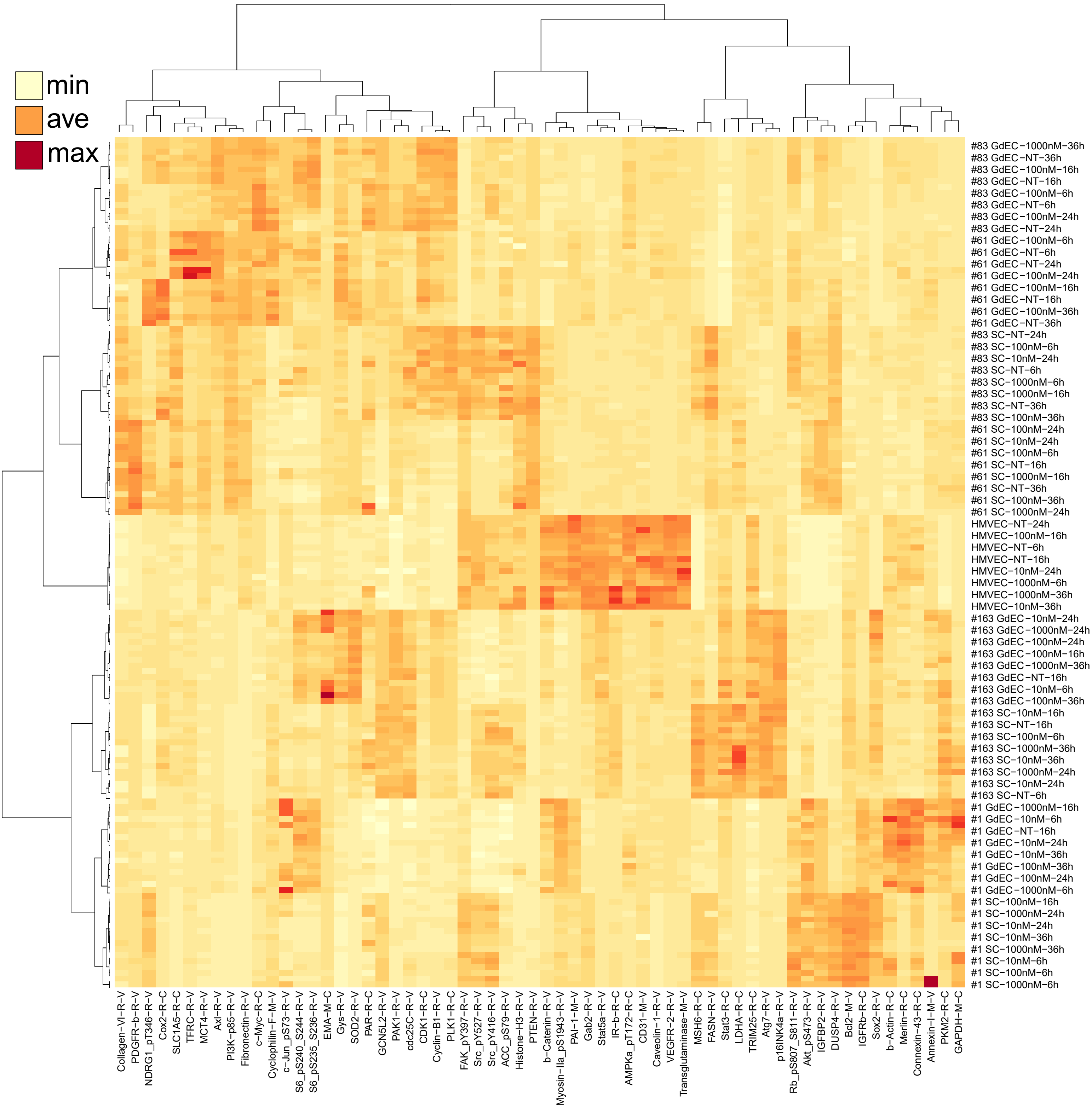

Supplementary Figure S8. Heatmap of the most important antibodies in each of the **k = 6** metagenes resulting from the model.

A

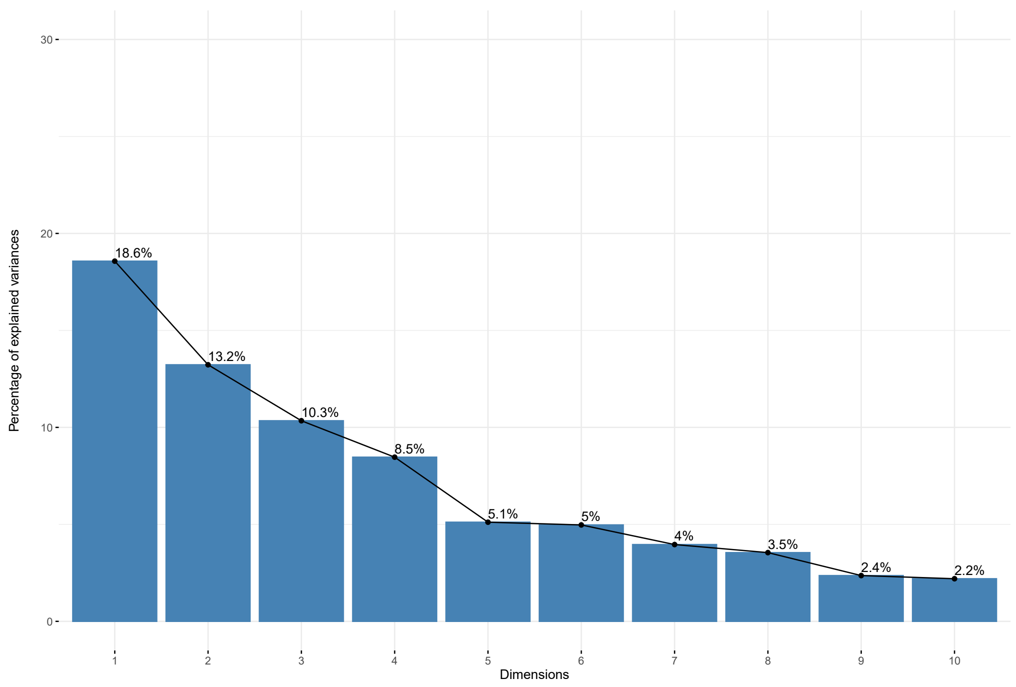

B

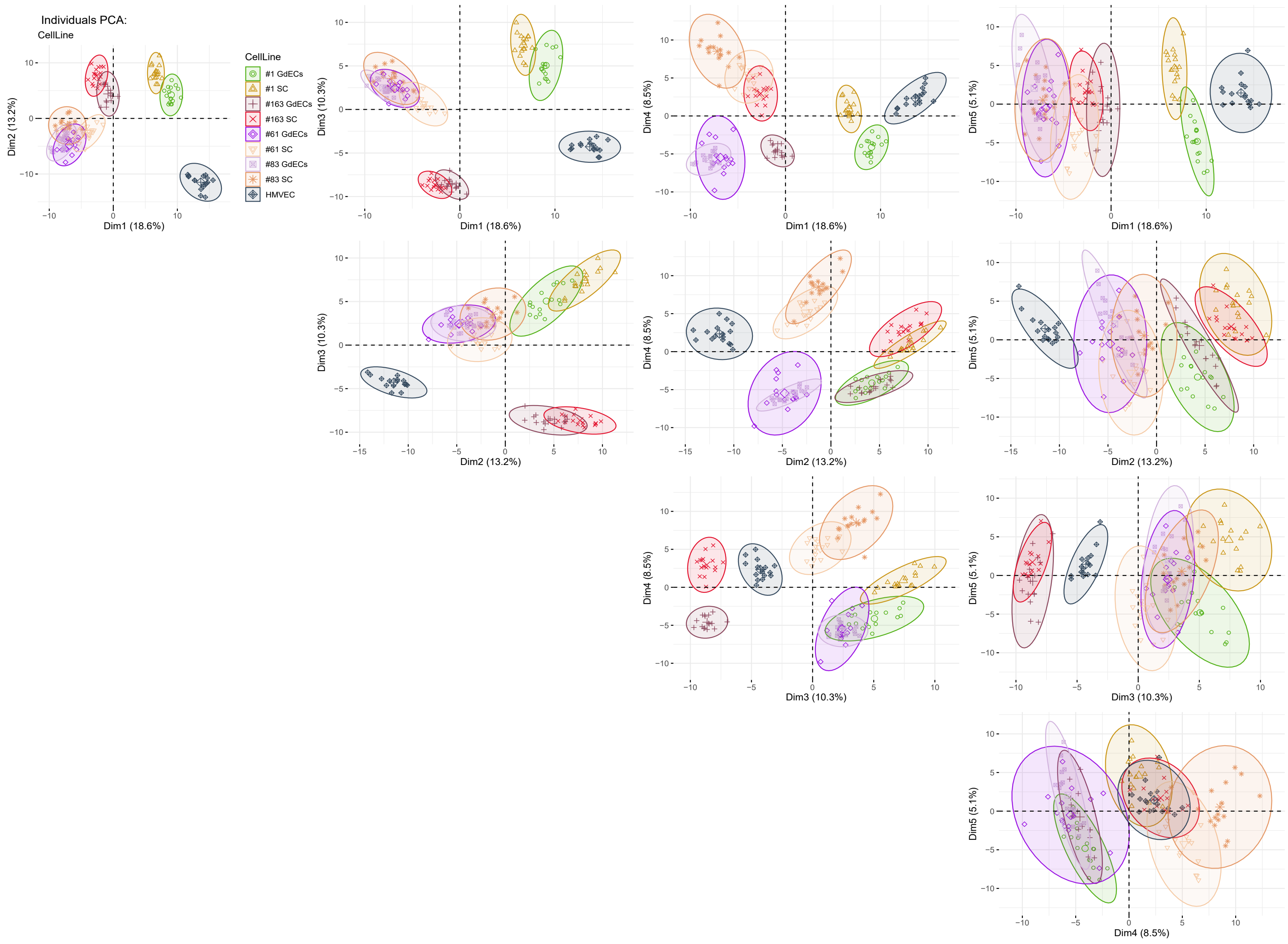

Supplementary Figure S9. Principal Component Analysis (PCA) algorithm applied to Elesclomol data, whereby each cell line is considered as a function of the antibodies. **A**) Scree plot. Given the low amount of variance explained by the variables above the fifth, we considered up to 5 principal components. **B**) Biplots using cell lines and growth conditions as scores. Ellipses represent the 95% probability of finding sample score values.

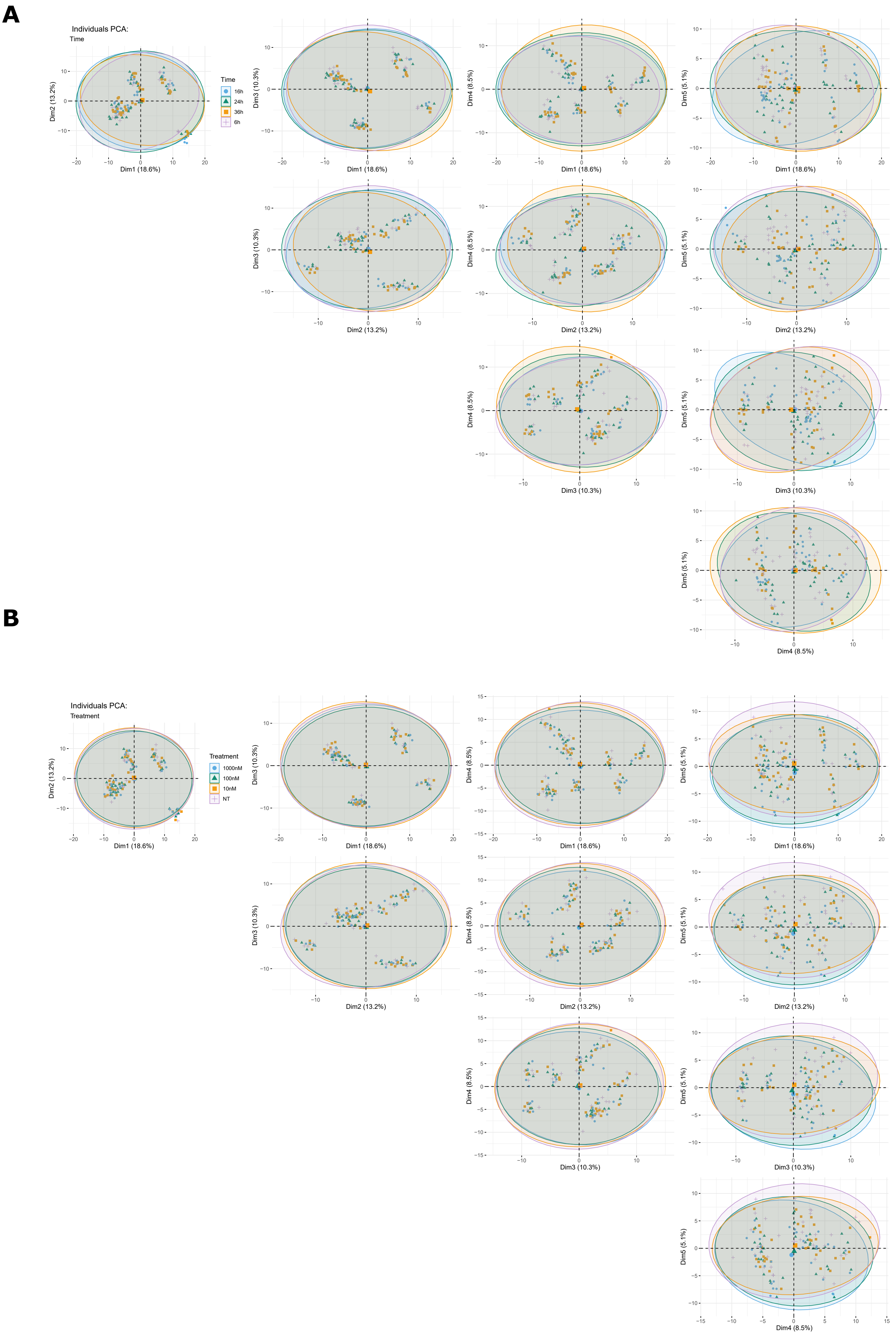

Supplementary Figure S10. Principal Component Analysis (PCA) biplots of components of the antibodies using (**A**) Time and (**B**) treatment, respectively. Ellipses represent the 95% probability of finding sample score values.

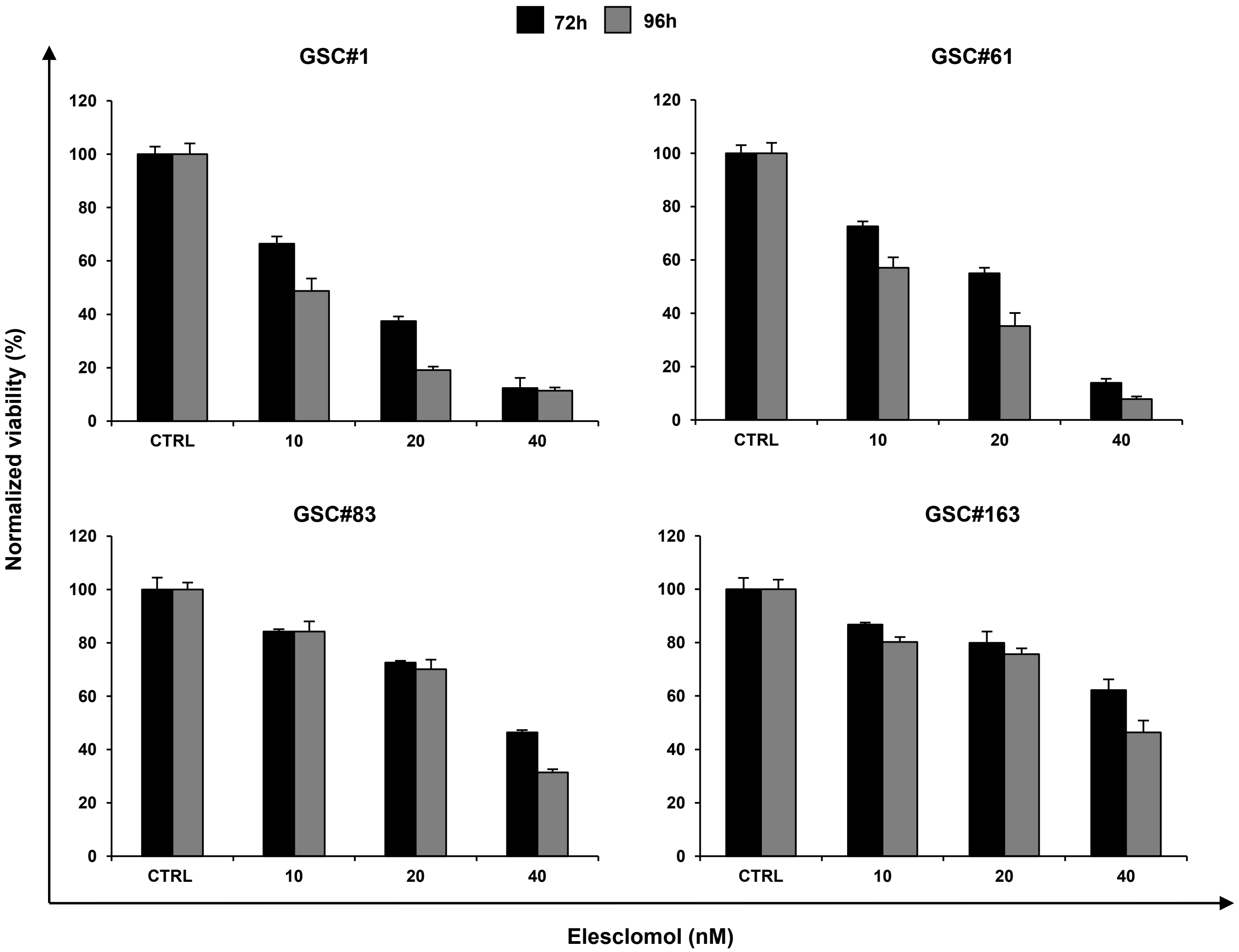

Supplementary Figure S11. Concent ration-response assays on all the four selected GSC lines for setting the dose of Elesclomol most suitable for the combination with TMZ.
